# Metabolic Adaptation of CD8⁺ T Cells Limits the Efficacy of Fatty Acid Oxidation Inhibition in Type 1 Diabetes

**DOI:** 10.7150/ijbs.125649

**Published:** 2026-01-30

**Authors:** Manuel Salzmann, Laura Boccuni, Patrizia Gibler, Mira Brekalo, Tamara S. Trimmel, Elena T. Pichler, Patrick Haider, Julia L. Blesch, Christian Hengstenberg, Michael B. Fischer, Bruno K. Podesser, Remi J. Creusot, Julia B. Kral-Pointner, Philipp J. Hohensinner

**Affiliations:** 1 Department of Internal Medicine II/Cardiology, Medical University of Vienna, Währinger Gürtel 18-20, 1090 Vienna, Austria.; 2 Ludwig Boltzmann Institute for Cardiovascular Research, Währinger Gürtel 18-20, 1090 Vienna, Austria; 3 Center for Biomedical Research and Translational Surgery, Medical University of Vienna, Währinger Gürtel 18-20, 1090 Vienna, Austria.; 4 Department of Transfusion Medicine and Cell Therapy, Medical University of Vienna, Vienna, Austria.; 5 Columbia Center for Translational Immunology, Department of Medicine and Naomi Berrie Diabetes Center, Columbia University Irving Medical Center, New York, NY, United States.

**Keywords:** fatty acid oxidation, metabolic reprogramming, type 1 diabetes, CD8^+^ T cell, Trimetazidine, CPT1A

## Abstract

Type 1 Diabetes Mellitus (T1D) is an organ-specific autoimmune disease characterized by persistent hyperglycemia due to immune-mediated destruction of pancreatic islet β-cells. Targeting immune cell metabolism has emerged as a promising therapeutic strategy. We investigated whether the fatty acid oxidation (FAO) inhibitor trimetazidine (TMZ), one of only three approved drugs directly targeting cellular metabolism, can restrain autoreactive immunity and delay T1D in non-obese diabetic mice (NOD). TMZ enhanced mitochondrial membrane potential, suppressed FAO, and curtailed activation and proliferation of human CD8^+^ T cells. In dysglycemic NOD mice, a clinically approved dose of TMZ delayed progression to T1D, reduced mean glycemia, and decreased islet CD4⁺/CD8⁺ infiltration. Single-cell RNA sequencing revealed depletion of FAO-high, stress-responsive cells and mitochondrially active stromal cells, indicating improved pancreatic health. Prolonged exposure induced compensatory upregulation of carnitine-palmitoyl-transferase-1A (CPT1A) in CD8⁺ subsets, counterbalancing early benefits. In summary, TMZ transiently restrains CD8⁺ T cell activity, reduces islet infiltration, and improves pancreatic health. The adaptive upregulation of CPT1A demonstrates a novel evasion mechanism to FAO inhibition and underscores the central role of FAO in sustaining pathogenic T cells. Our work highlights metabolic adaptation as a key determinant of autoimmune progression, validating FAO as a therapeutic target in T1D.

## Introduction

Type 1 diabetes (T1D) is a chronic autoimmune disease characterized by immune-mediated destruction of pancreatic β-cells, ultimately leading to life-long insulin dependence. The disease develops in distinct stages, beginning with islet autoimmunity, progressing to dysglycemia, and culminating in overt diabetes 1. A central feature of T1D pathogenesis is the breakdown of immune tolerance, with autoreactive T cells recognized as the major drivers of β-cell destruction. Among these, CD8⁺ cytotoxic T lymphocytes directly infiltrate pancreatic islets and mediate β-cell killing, while CD4⁺ T helper cells amplify autoimmunity by promoting pro-inflammatory responses and providing help to B cells and other immune effectors 2. The critical role of T cells in disease development is underscored by the recent clinical success of immune interventions such as teplizumab, an anti-CD3 antibody, which delays disease onset in at-risk individuals 3. These findings highlight T cells not only as key effectors in T1D but also as promising therapeutic targets for interventions aimed at modifying the natural history of the disease.

T cell activation and differentiation are tightly coupled to metabolic reprogramming, enabling these cells to meet the energetic and biosynthetic demands of immune responses. Effector CD8⁺ T cells rely predominantly on glycolysis to sustain rapid proliferation and cytotoxic activity, whereas long-lived memory T cells depend more on mitochondrial fatty acid oxidation (FAO) to maintain persistence and function 4. This metabolic plasticity is not merely a byproduct of activation but a critical determinant of T cell fate and function. Consequently, interventions that modulate cellular metabolism offer a promising strategy to alter immune responses in autoimmunity. Indeed, experimental evidence has shown that enforced glycolysis can impair CD8⁺ T cell memory formation, while disruption of FAO perturbs memory differentiation 5, 6. Given the central role of CD8⁺ T cells in β-cell destruction during T1D, targeting their metabolic pathways represents a rational therapeutic approach to restrain autoreactive immunity and potentially delay disease progression.

Metabolic modulators are drugs that alter cellular or systemic metabolism to treat disease. Despite their potential, only three agents directly target core metabolic pathways such as glycolysis, fatty acid oxidation (FAO), oxidative phosphorylation (OXPHOS), and the tricarboxylic acid (TCA) cycle have received clinical approval. The most prominent metabolic modulator is metformin used as a first in line treatment option in Type 2 Diabetes (T2D). Metformin modulates gluconeogenesis and inhibits OXPHOS by binding to complex I, ultimately lowering blood glucose levels 7, 8. Two further drugs, trimetazidine (TMZ) and perhexiline, inhibit FAO and are approved for the treatment of chronic stable angina and hypertrophic cardiomyopathy respectively 9, 10. However, the clinical trial of etomoxir, the most commonly used inhibitor of FAO in experimental models, was prematurely terminated due to unacceptably high levels of liver transaminases 11. This hepatotoxicity is also shared by perhexiline 12, while TMZ is not associated with liver damage 13.

Its favorable safety profile made TMZ a well-studied and clinically established partial FAO inhibitor 14. It is widely used to treat angina and recommended as an adjunct to β-blockers or calcium antagonists 13. TMZ partially inhibits 3-ketoacyl-CoA thiolase (HADHB), a key enzyme in the β-oxidation pathway 15, shifting cellular metabolism from FAO toward glycolysis 16, enhancing cell viability 17 and exhibiting anti-inflammatory 18, antioxidative, and mitoprotective properties 19. Positive effects of TMZ treatment were also evident in patients with T2D 20, 21. Despite its widespread use in cardiovascular therapy, long-term outcome data from the ATPCI (“A Study to Assess the Efficacy and safety of Trimetazidine in patients with angina pectoris treated by Percutaneous Coronary Intervention”) study revealed no reduction in recurrent cardiac events or mortality 13, suggesting its primary benefits may be limited to symptomatic improvement 22-26.

Using primary human T cells and the non-obese diabetic (NOD) mouse model of T1D 27, 28, we investigated whether TMZ could modulate T cell metabolism to delay disease onset. We found that TMZ treatment of dysglycemic mice delayed diabetes progression, reduced T cell infiltration, and improved pancreatic health. However, prophylactic TMZ treatment had no effect. *In vitro*, TMZ transiently impaired CD8^+^ T cell memory differentiation, an effect that correlated with compensatory upregulation of carnitine palmitoyltransferase 1A (CPT1A), the rate-limiting enzyme of FAO. Our findings suggest that while TMZ offers a safe and initially effective strategy for modulating immune metabolism, cellular adaptation may limit its long-term efficacy.

## Material and Methods

### Mice and T1D model

We purchased 140 five-week-old female NOD/ShiLtJ mice (Strain #:001976) from The Jackson Laboratory (Bar Harbor, ME, USA). Five mice were euthanized early based on humane endpoints and excluded from the experiments. Mice were housed under specific pathogen-free conditions at the Core Facility Laboratory Animal Breeding and Husbandry, Medical University of Vienna, in individually ventilated cages (maximum five per cage) with dust-free bedding, nesting material, and ad libitum access to autoclaved tap water and pelleted food. Environmental conditions were maintained at 21 ± 2 °C, 55 ± 10% humidity, with a 12:12-hour light-dark cycle. Cage changes were performed in a biosafety cabinet, and mice were euthanized by cervical dislocation.

Fasting blood glucose was measured from lateral tail vein puncture (25G needle) after 4-5 hours of morning fasting using a Contour Next glucometer (Ascensia Diabetes Care, Vienna, Austria). Measurements were taken twice weekly in the intervention experiment and once weekly in the prophylaxis setting. Mice were enrolled when fasting glucose reached 150-180 mg/dL and received 62.5 mg/L Trimetazidine dihydrochloride (MedChemExpress, NJ, USA) in drinking water (approx. 15 mg/kg/day, in adaption to the human maximum dose of 70 mg/day 18, 29). In the prophylaxis experiment, mice received TMZ or control water starting at 5 weeks of age. Diabetes was defined as fasting glucose > 200 mg/dL for one week, upon which mice were euthanized.

### Human T cell culture

Peripheral blood T cells were isolated from apheresis chambers of healthy donors with informed consent and in accordance with ethical approval EK 1575/2014 from the Medical University of Vienna. Cell isolation and activation were performed using commercial kits from STEMCELL Technologies (Stolberg, Germany) following the manufacturer's protocols: EasySep Human T Cell Isolation Kit, Naïve CD4^+^ T Cell Isolation Kit II, Naïve CD8^+^ T Cell Isolation Kit II, and ImmunoCult Human CD3/CD28/CD2 T Cell Activator. A total of 36 individual human donors were used.

Cells were cultured in RPMI 1640 medium (1.8 - 2.2 g/L glucose) (Sigma-Aldrich, St. Louis, MO, USA) supplemented with 2 mM L-glutamine and Antibiotic-Antimycotic (Thermo Fisher Scientific, Waltham, MA, USA), 10% FBS Gold (Seraglob, Schaffhausen, Switzerland), and 10 ng/mL recombinant human Interleukin (IL)-2 (BioLegend, Amsterdam, The Netherlands). TMZ-dihydrochloride (500 µM; MedChemExpress) or vehicle (water) was added as indicated. For long-term cultures, media were refreshed every other day with IL-2 supplementation.

### Mitochondrial function, ROS, viability, activation, and proliferation assays

Mitochondrial membrane potential was assessed using the JC-1 Assay Kit (Abcam, Cambridge, UK) following the manufacturer's instructions. Mitochondrial and cytosolic ROS were measured using MitoSOX Red (1:1000 in PBS) and CellROX Green (1:500 in RPMI), respectively (both Thermo Fisher Scientific). Cell viability was determined with Annexin V APC (1:100, BioLegend) and 7-AAD (1:100, Thermo Fisher Scientific) in Annexin V Binding Buffer (Thermo Fisher Scientific). CD25 BV650 (2 µg/mL, clone BC96, BioLegend) and Ki-67 FITC (12 µg/mL, clone 20Raj1, Thermo Fisher Scientific) were used after fixation and permeabilization with ice-cold methanol to assess T cell activation and proliferation. Samples were acquired on an Attune NxT Flow Cytometer and analyzed using Attune NxT Software v3.1.2 (Thermo Fisher Scientific). FAO was measured using the Extracellular Oxygen Consumption Assay (Abcam) in complete medium. 10 µM Etomoxir (TargetMol, Linz, Austria), a CPT1A inhibitor, was added 10 minutes prior to measurement to block mitochondrial import and oxidation of long-chain fatty acids; FAO-dependent oxygen consumption was calculated as the difference from total Oxygen Consumption Rate (OCR).

### EndoC-βH5 culture

EndoC-βH5 human pancreatic β-cells (Human Cell Design, Toulouse, France) were cultured in βcoat-coated plates using ULTIβ1 medium (both from Human Cell Design). Glucose-stimulated insulin secretion (GSIS) was performed with ULTI-ST and βKREBS medium (Human Cell Design), 20 mM glucose, and 500 µM TMZ for 40 minutes. Insulin levels were measured using the Human Insulin ELISA Kit (10-1113-01, Mercodia, Uppsala, Sweden) and analyzed according to the manufacturer's instructions.

For inflammatory stimulation, EndoC-βH5 cells were cultured for 6 days in ULTIβ1 medium, followed by treatment with 20 ng/mL human Interferon (IFN) γ (Thermo Fisher Scientific) and/or 500 µM TMZ for 48 hours.

### qPCR

For EndoC-βH5, RNA was extracted using the Monarch Total RNA Miniprep Kit (New England Biolabs (NEB), Ipswich, MA, USA) and reverse transcribed into cDNA using LunaScript RT Supermix (NEB). Gene expression levels of *CXCL10* and *HLA-B* were quantified using the 2^-ΔΔCt method, normalized to 18S rRNA as a housekeeping gene and to the mean expression level of untreated control cells.

For qPCR of pre-onset and diabetic pancreatic tissue, all samples from 10 weeks of prophylactic TMZ treatment were analyzed. RNA was isolated using Maxwell® RSC simplyRNA Tissue Kit (Promega), in combination with a ball mill, and reverse transcribed into cDNA using LunaScript RT Supermix (NEB). Gene expression levels of *Cpt1a, Idh2, Prdx2,* and *Slc2a1* (Glut1) were quantified using the 2^-ΔΔCt method, normalized to 18S rRNA as a housekeeping gene and to the mean expression level of respective untreated control tissue.

Quantitative PCR (qPCR) was performed using a C1000 Touch Thermal Cycler (Bio-Rad) with Luna Universal qPCR Master Mix (NEB). Primer sequences are provided in **Table 1.**

### Flow cytometry

For murine samples, 5 µL of citrated whole blood was stained with anti-mouse antibodies for 15 minutes at room temperature. After staining, samples were fixed in 1% formaldehyde for 10 minutes, followed by erythrocyte lysis in a buffer containing 150 mM NH_4_Cl, 10 mM KHCO_3_, and 0.1 mM Na_2_EDTA for 5 minutes. Pancreatic lymph nodes were mechanically dissociated through a 70 µm cell strainer, washed with PBS, and 10 µL of single-cell suspension was stained for 15 minutes. Fixation and permeabilization were performed overnight using the Foxp3/Transcription Factor Staining Buffer Set (Thermo Fisher Scientific), followed by intracellular staining. Murine samples were acquired on an Attune NxT Flow Cytometer and analyzed using Attune NxT Software v3.1.2 (Thermo Fisher Scientific). Murine T cell gating: naïve (T_N_; CD44^low^CD62L^high^), pre-effector (T_PE_; CD44ˡᵒʷCD62Lˡᵒʷ) effector memory (T_EM_; CD44^high^CD62L^low^), and central memory (T_CM_; CD44^high^CD62L^high^).

For human samples, cultured T cells were stained with anti-human surface antibodies for 15 minutes at room temperature. For intracellular metabolic staining, cells were fixed and permeabilized using the Foxp3/Transcription Factor Staining Buffer Set (Thermo Fisher Scientific) according to the manufacturer's instructions, followed by intracellular staining. Human samples were acquired on a BD FACSymphony A1 flow cytometer and analyzed using FlowJo X software (BD Biosciences, San Jose, USA). Human T cell gating: Naïve (T_N_; CCR7⁺CD45RA⁺), central memory (T_CM_; CCR7⁺CD45RA⁻), effector memory (T_EM_; CCR7⁻CD45RA⁻), and terminally differentiated effector memory T cells re-expressing CD45RA (T_EMRA_; CCR7⁻CD45RA⁺).

The antibodies used for murine and human flow cytometry are listed in **Table 2**.

### Histology

Pancreas samples were fixed in 4% formaldehyde, embedded in paraffin, and sectioned at 3 µm thickness. For immunofluorescence staining, sections were deparaffinized, rehydrated, and subjected to antigen retrieval using either citrate buffer (10 mM citrate, 0.05% Tween-20, pH 6.0) or Tris-EDTA buffer (10 mM Tris, 1 mM EDTA, 0.05% Tween-20, pH 9.0). Blocking was performed for 90 minutes at room temperature using a solution containing 2% bovine serum albumin, 0.5% fish gelatin, and 0.3% Tween-20. Primary antibodies were applied overnight at 4 °C, followed by incubation with the corresponding secondary antibodies for 2 hours at room temperature. DAPI (1 µg/mL) was used for nuclear counterstaining. Antibodies and antigen retrieval conditions are listed in **Table 3**. Fluorescence imaging was performed using an automated TissueFAXS microscopy stage on a Zeiss Observer Z1 microscope (NA 0.5) at 20× magnification.

### Image analysis

Image analysis was conducted as previously described in a comparable workflow 30-32, with adaptations for pancreatic tissue and antibody panels. Fluorescence images were imported into QuPath 33, and islets of Langerhans were manually annotated by two researchers blinded for control and treatment groups. Islet cells were detected using QuPath's “Cell detection” feature, with a DAPI threshold of 1000 thereby excluding autofluorescence from erythrocytes. Cell expansion was adjusted according to subcellular localization of the respective marker. After cell detection, intensity parameters were exported, and two researchers jointly determined the optimal positivity threshold for each channel based on the images.

Each data point represents a single slide from one mouse, corresponding to one pancreatic cross-section. Cell counts were normalized to the area of their respective regions in mm². If no islets were detected on the initial slide, a second tissue section from the same pancreas was stained. If no islets were found in either section, the sample was excluded from analysis due to absence of islet tissue. The average of analyzed islet tissue area per mouse (i.e., the total islet area per slide) and the mean number of detected cells per slide are reported in the corresponding figure legends.

### Pancreas single cell RNA sequencing

Pancreatic tissue was collected from five control mice and five TMZ-treated mice after one week of treatment, initiated when fasting blood glucose reached 150-180 mg/dL. Pancreatic heads were dissected, snap-frozen, and stored in liquid nitrogen until processing. Tissue dissociation and fixation were performed using the Chromium Next GEM Single Cell Fixed RNA Sample Preparation Kit (PN-1000414, 10x Genomics, Pleasanton, CA, USA). Nuclei and single cells were isolated following the protocol “Tissue Fixation & Dissociation for Chromium Fixed RNA Profiling” (CG000553). Samples from each group (control and TMZ-treated) were pooled and assigned one barcode per group for multiplexed analysis.

Library preparation was performed using the “Chromium Fixed RNA Profiling for Multiplexed Samples” protocol (CG000527, protocol version F), with the Chromium Fixed RNA Profiling Kit - Mouse Transcriptome (4 reactions × 4 barcodes, PN-1000496), Hybridization & Library Kit (PN-1000415), and the Chromium Next GEM Chip Q Single Cell Kit (16 reactions, PN-1000422), all from 10x Genomics. Sequencing was performed on an Illumina NextSeq 2000 instrument using a NextSeq 2000 P4 flow cell (100 cycles, PN-20100994, Lot #20879630, Illumina, San Diego, CA, USA).

Sequencing data were demultiplexed and aligned using Cell Ranger software version 8.0.1 (10x Genomics), with the *Mus musculus* mm10 (Ensembl 98). Cell Ranger Loupe Browser output files were imported into R (version 4.4.1) for downstream processing. Nuclei with > 45% mitochondrial transcript content, nFeature_RNA ≤ 500, or nCount_RNA ≥ 500 were excluded from analysis. Filtered data were normalized, log-transformed, and the resulting matrix was used for all downstream analyses.

Data integration was performed using RPCA-based batch correction implemented in Seurat v5 (https://satijalab.org/seurat/articles/seurat5_integration). Principal component analysis was conducted using the first 20 components, and clustering was performed with a resolution of 0.1. The final annotated dataset was exported as a cloupe file and reloaded into the Loupe Browser (10x Genomics) for visualization and manual inspection of clusters. Pseudo-bulk differential expression analysis was performed using the DElegate package (https://github.com/cancerbits/DElegate). Cluster identities and enriched pathways were inferred by uploading significantly upregulated genes (adjusted p < 0.05, log₂ fold change > 1) to Enrichr 34-36. Statistical analysis of the difference between cell proportion in clusters between TMZ and control samples was performed with scProportionTest package (https://github.com/rpolicastro/scProportionTest; 37). Heatmap of clusters was generated with Clustergrammer 38, and volcano plots were produced using VolcaNoseR 39.

### EndoC-βH5 bulk RNA sequencing

Sequencing libraries from total RNA were prepared at the Core Facility Genomics, Medical University of Vienna, using the QuantSeq 3′ FWD protocol version 2 with unique dual indices (Lexogen, Greenland, NH, USA), following the low input branch of the manufacturer's instructions. 20 PCR cycles were used for library amplification. PCR cycle numbers were determined by qPCR according to the library preparation manual.

Libraries were quality-checked using a Bioanalyzer 2100 (Agilent, Santa Clara, CA, USA) with a High Sensitivity DNA Kit to confirm insert size and quantified using the Qubit dsDNA HS Assay (Thermo Fisher). Pooled libraries were sequenced on a NextSeq 2000 system (Illumina) using a P2 flow cell in single-end 1×75 bp mode.

Reads were demultiplexed using Illumina bcl2fastq (v2.19.1.403) and the Lexogen *idemux* tool (https://github.com/Lexogen-Tools/idemuxcpp) for optimized handling of long unique dual indices. Adapter and polyA trimming, base quality filtering (Q<30), and removal of reads containing ambiguous bases (N) were performed using *cutadapt* 40 version 2.8. On average, 9 million raw reads and 2.5 million filtered reads were obtained per sample from EndoC-βH5 cells.

Trimmed reads were aligned to the human reference genome GRCh38 41 with Gencode v29 annotation 42 using STAR aligner (v2.6.1a) 43 in 2-pass mode. Gene-level raw counts were generated with STAR. Differential gene expression was analyzed using DESeq2 version 1.22.2 44.

### Statistics

Statistical analyses were performed using GraphPad Prism version 8.0 (GraphPad Software, Boston, MA, USA). All tests were two-sided. Paired data from *in vitro* experiments were analyzed using paired statistical tests. *In vivo* data were tested for normality using the Anderson-Darling test. Data with Gaussian distribution were analyzed using unpaired t-tests; non-Gaussian data were assessed using the Mann-Whitney test.

Time to diabetes onset was evaluated using Kaplan-Meier survival analysis and compared with the Gehan-Breslow-Wilcoxon test. The hazard ratio was calculated using the Mantel-Haenszel method. Blood glucose curves were analyzed using a two-way ANOVA mixed-effects model with Geisser-Greenhouse correction. Reported p-values reflect the time × treatment interaction. Sample sizes are indicated in the respective figure legends.

## Results

### Trimetazidine modulates mitochondrial function, reduces activation, and impairs proliferation in human T cells

Chronic disease conditions promote mitochondrial, OXPHOS-dependent metabolism in T cells, whereas acute inflammation is associated with glucose-dependent metabolism 4. We were interested in the extent to which FAO inhibition by TMZ during T cell receptor activation would alter overall T cell function *in vitro*. Total CD3^+^ T cells from healthy human donors were isolated and incubated with ImmunoCult CD3/CD28/CD2 T cell Activator, supplemented with IL-2 and TMZ treatment or vehicle control.

**Figure 1:**
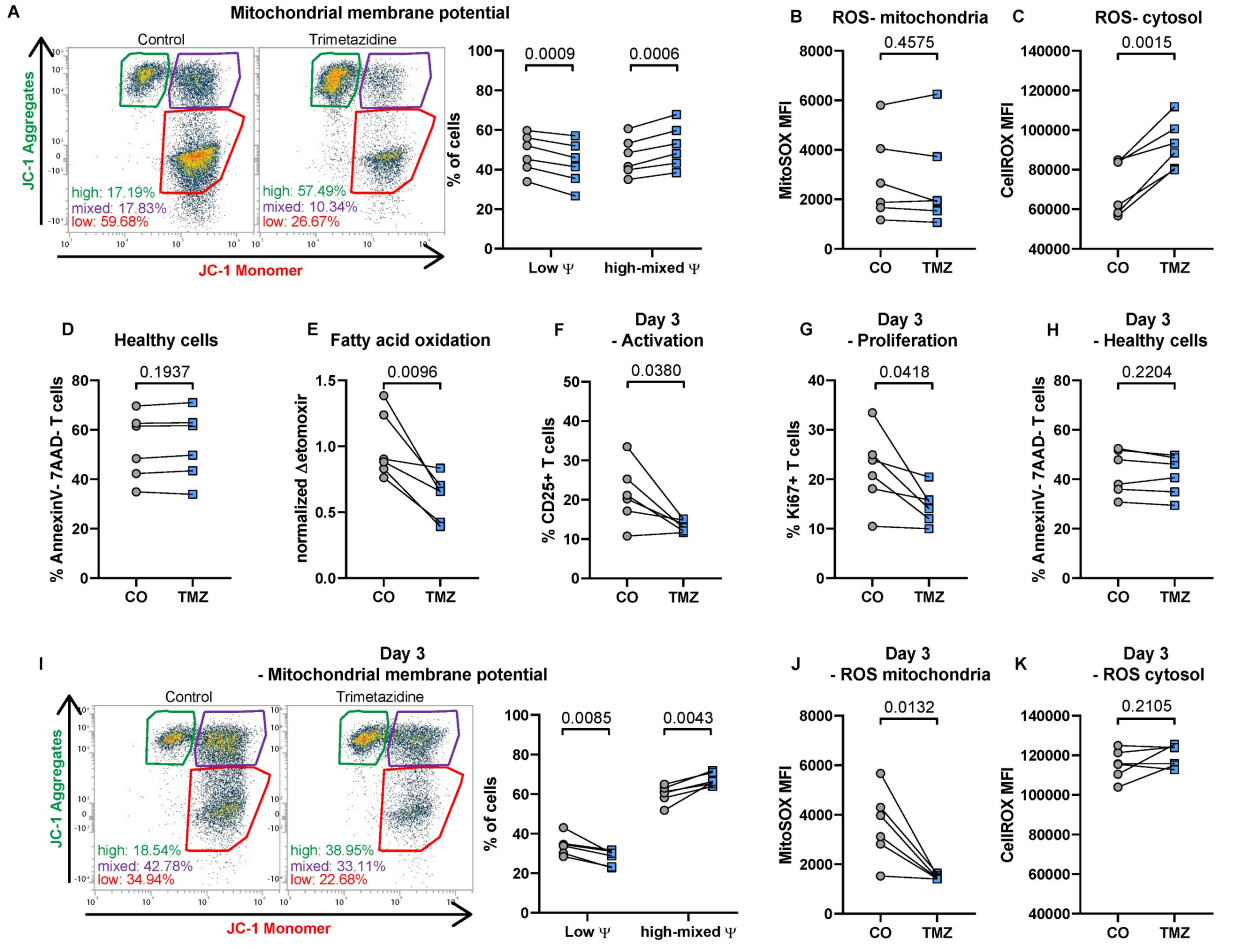
** Trimetazidine modulates mitochondrial function, reduces activation, and impairs proliferation in human T cells.** (A) Total human T cells were stimulated with anti-CD3, CD28, and CD2 with or without TMZ for 24 hours and analyzed for mitochondrial membrane potential using JC-1 dye. Gates indicate populations with low membrane potential (monomers), high membrane potential (aggregates), and intermediate states. (B) Mitochondrial and (C) total reactive oxygen species (ROS) levels. (D) Cell viability, defined as Annexin V^-^/7-AAD^-^ cells. (E) Fatty acid oxidation (FAO), measured via oxygen consumption rate and compared to the FAO inhibitor etomoxir. (F) CD25 surface expression and (G) intracellular Ki67 expression were assessed after 72 hours of TMZ treatment. (H) Cell viability (Annexin V^-^/7-AAD^-^) after 72 hours. (I) Mitochondrial membrane potential measured with JC-1 dye after 72 hours. (J) Mitochondrial and (K) total ROS levels after 72 hours of TMZ. Data represents n = 6 donors. Paired t-tests were used for statistical analysis. CO: Control; TMZ: Trimetazidine; φ: mitochondrial membrane potential.

After 24 hours, TMZ significantly increased mitochondrial membrane potential, reflected by a shift in the proportion of cells with high potential, indicating enhanced mitochondrial function 45 (**Fig. 1A**). Mitochondrial ROS levels, however, remained unaltered (**Fig. 1B**), while total ROS levels increased following TMZ exposure (**Fig. 1C**). Importantly, cell viability remained stable (**Fig. 1D**). Functionally, FAO, assessed via oxygen consumption using the CPT1 inhibitor etomoxir as an indicator for complete FAO inhibition 46, was markedly suppressed in TMZ-treated cells (**Fig. 1E**).

When analyzing T cells three days after initial activation with or without TMZ treatment we observed reduced T cell activation indicated by reduced CD25 expression (**Fig. 1F**). In addition, T cell proliferation was diminished at the 72-hour time point in the TMZ group compared to control T cells (**Fig. 1G**). Already 50 µM TMZ was able to reduce T cell proliferation (43.42 ± 3.22 % in controls vs 39.79 ± 2.39 % with 50 µM TMZ, n = 6, p = 0.0157, paired t test). Cell viability remained stable (**Fig. 1H**), and mitochondrial membrane potential remained elevated in TMZ-treated T cells compared to controls (**Fig. 1I**). This was accompanied by a substantial reduction in mitochondrial ROS levels in TMZ-treated cells, while control cells maintained high mitochondrial ROS levels three days after activation (**Fig. 1J**). Conversely, total ROS remained stable in TMZ-treated cells but increased in control cells over time, reaching levels similar to those induced by TMZ at day 1 (**Fig. 1K**). Our data therefore indicate that FAO inhibition reduces overall T cell activation.

### Trimetazidine intervention delays diabetes onset and reduces islet T cell infiltration in dysglycemic NOD mice

**Figure 2:**
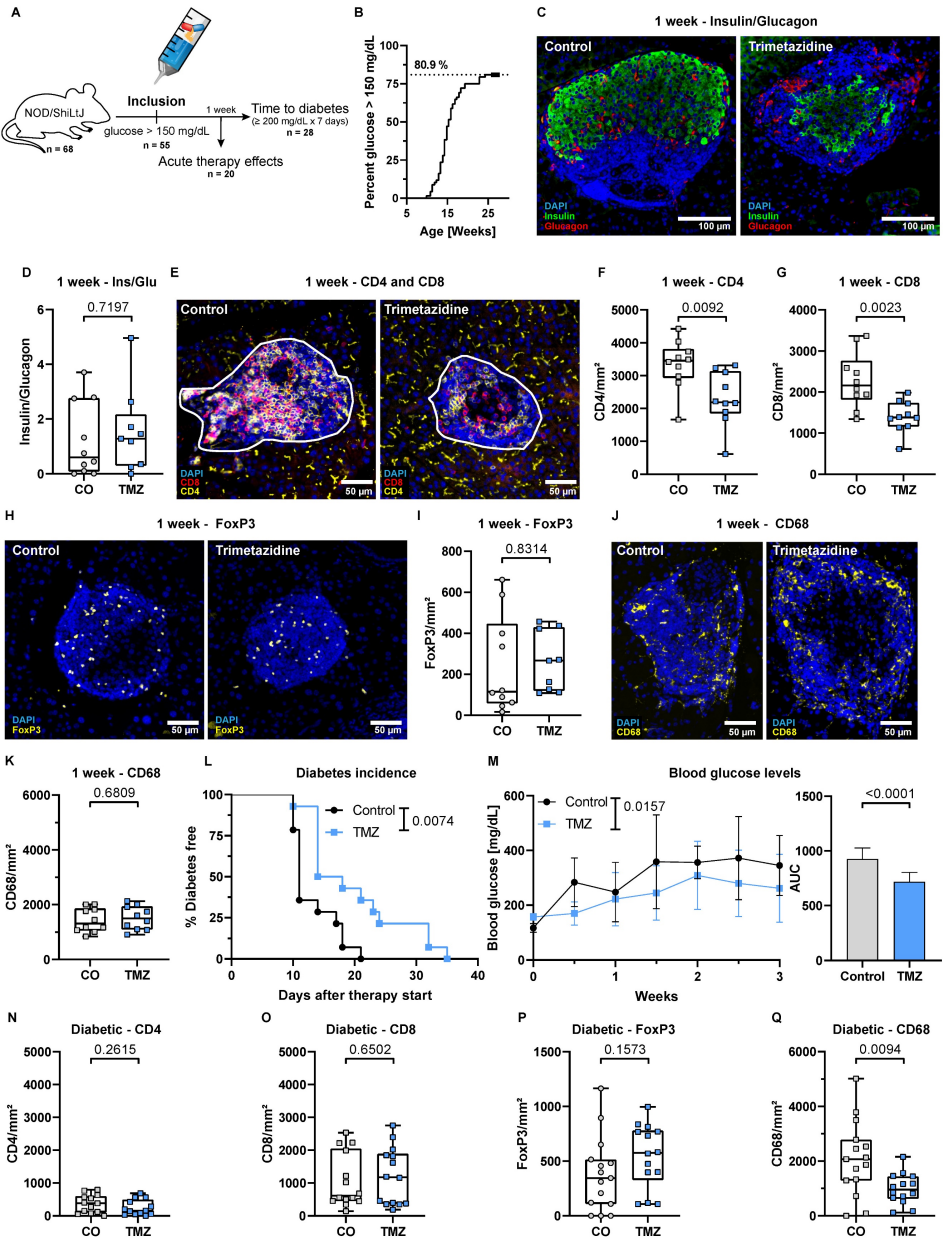
** Trimetazidine delays diabetes onset and reduces islet T cell infiltration in dysglycemic NOD mice.** (A) Schematic overview of the experiment, starting with 68 female NOD/ShiLtJ mice. Of these, 55 mice reached a fasting blood glucose of 150 mg/dL but no more than 180 mg/dL and were treated with TMZ in drinking water. 20 randomly allocated mice sacrificed after 1 week, while the remaining 35 were followed until diabetes onset. In total, 28 of these mice developed diabetes, defined as fasting blood glucose ≥200 mg/dL sustained for one week, measured twice per week. (B) Proportion of colony animals enrolled in the study over time. n= 80. (C) Representative images showing β-cell (insulin) and α-cell (glucagon) distribution in dysglycemic mice. (D) Quantification of β-cell (insulin) to α-cell (glucagon) ratios in pancreatic islets after 1 week of treatment. Mean total islet area analyzed per mouse: 137,460 µm². n= 9-10. (E) Representative images of CD4^+^ and CD8^+^ T cell infiltration in pancreatic islets (white outlines). (F-G) Quantification of CD4^+^ and CD8^+^ T cell infiltration into pancreatic islets. Mean total islet area analyzed per mouse: 234,377 µm². n= 10. (H) Representative images of FoxP3^+^ regulatory T cells. (I) Quantification of FoxP3^+^ regulatory T cells in pancreatic islets. Mean total islet area analyzed per mouse: 137,460 µm². n= 9-10. (J) Representative images CD68^+^ macrophages. (K) Quantification of CD68^+^ macrophage in pancreatic islets. Mean total islet area analyzed per mouse: 125,565 µm². n= 10. (L) Kaplan-Meier survival curve showing delayed diabetes onset in TMZ-treated mice following intervention. P-value calculated with the Gehan-Breslow-Wilcoxon test. n= 14. (M) Fasting blood glucose levels during the 3-week follow-up, with area under the curve (AUC) comparison. Two-way ANOVA mixed-effects model with Geisser-Greenhouse correction; p-value reflects the time x treatment interaction. (N-Q) Quantification of (N) CD4^+^ (N), CD8^+^ (O), regulatory T cells (P), and macrophages (Q) infiltration in diabetic mice treated with TMZ. Mean total islet area analyzed per mouse: (N-O) 74,548 µm². n= 13-14; (P) 65,402 µm². n= 14; (Q) 95,811 µm². n= 14. Unpaired t-test or Mann-Whitney test were used for statistical analysis. CO = Control, TMZ = Trimetazidine.

T cells are key effector cells in the autoimmune destruction of pancreatic β-cells in T1D. To evaluate the therapeutic potential of TMZ in this context, we conducted an experiment with 68 female NOD/ShiLtJ mice, which spontaneously develop T cell-mediated T1D (**Fig. 2A**). Of these, 55 mice (80.9%) reached an elevated fasting blood glucose level of > 150 mg/dL (**Fig. 2B**), similar to patients showing symptomatic T1D signs at stage 2 of the disease 1, 47, 48. These mice were randomly selected to receive TMZ or vehicle control via the drinking water after inclusion into the experiment. 20 mice were randomly allocated and sacrificed after one week of treatment (equal number of treated and control animals), while the remaining 35 were followed until diabetes onset. In total, 28 of these mice developed diabetes, defined as fasting blood glucose ≥ 200 mg/dL sustained for one week (**Fig. 2A**).

After one week of treatment, control mice had 279 ± 137 mg/dL and TMZ-treated mice 194 ± 92 mg/dL blood glucose. β-cell to α-cell ratio was unchanged, indicating similar insulin production capacities within the pancreas (**Fig. 2C-D**). Notably, TMZ reduced infiltration of both CD4^+^ and CD8^+^ T cells into pancreatic islets (**Fig. 2E-G**), suggesting that insulitis has been reduced in the one week of treatment following enrolment. The presence of regulatory T cells (Tregs) (**Fig. 2H-I**), associated with tissue protection in T1D, and islet macrophages (**Fig. 2J-K**) remained unchanged. In circulation, the frequencies of T cells, B cells, and neutrophils were unaffected, while monocyte counts were slightly increased without changes in subset distribution in TMZ-treated animals (**Fig. S1A-F**). Further phenotyping revealed no significant changes in circulating CD4^+^ and CD8^+^ T cell subsets (**Fig. S1G-H**). However, in pancreatic lymph nodes (PLN), a reduction in CD4^+^ T cells was observed (**Fig. S2A**), accompanied by an increase in CD8^+^CD25^+^ T cells (**Fig. S2B**).

TMZ treatment significantly delayed diabetes onset, with a median time to onset of 16 days in the TMZ group compared to 11 days in controls (**Fig. 2L**). This corresponded to a 1.5-fold increase in diabetes-free survival, with a Mantel-Haenszel hazard ratio of 0.2279 (95% CI: 0.084-0.615), indicating a 77% lower instantaneous risk of disease progression compared to controls. Overall, blood glucose levels remained significantly lower compared to control animals over the first three weeks (**Fig. 2M**). *In vitro*, TMZ reduced glucose-stimulated insulin secretion in the human β-cell line EndoC-βH5, (**Fig. S3A**), while bulk RNA sequencing revealed only minimal TMZ-induced transcriptional changes (**Fig. S3B**). Enriched gene sets may hint towards β-cell protection but were not associated with metabolic or stress responses (**Table S1**). Likewise, inflammatory responses to IFNγ in EndoC- βH5 remained unaffected (**Fig. S3C-D**).

When animals reached a diabetic state defined as 200 mg/dL glucose, the previously observed TMZ-induced reduction in CD4⁺ and CD8⁺ T cell infiltration was no longer detectable. CD4^+^ and CD8^+^ cell numbers within islets were similar for both the TMZ-treated group and the control group (**Fig. 2N-O**). Also, regulatory T cell infiltration was again comparable in both groups (**Fig. 2P**). Noteworthy, macrophage presence in the pancreas was reduced after diabetes onset (**Fig. 2Q**), together with a decline in circulating Ly6C^high^ monocytes by TMZ treatment (**Fig. S4E**). However, the numbers of circulating T cells, B cells, and neutrophils remained unchanged (**Fig. S4A-F**). Further immunophenotyping revealed only a slight increase in CD4^+^ pre-effector T cells in circulation but no changes in PLN in control animals versus TMZ-treated mice (**Fig. S4G-H, Fig. S5A-B**), suggesting that TMZ's early immunomodulatory effects had waned by the time of diabetes onset. This loss of effect at the diabetic stage suggests that the initial immunomodulatory benefit of TMZ is transient, and ultimately insufficient to prevent disease progression despite delaying its onset.

### Trimetazidine reduces mitochondrial and inflammatory stress signatures in the dysglycemic pancreatic tissue

**Figure 3:**
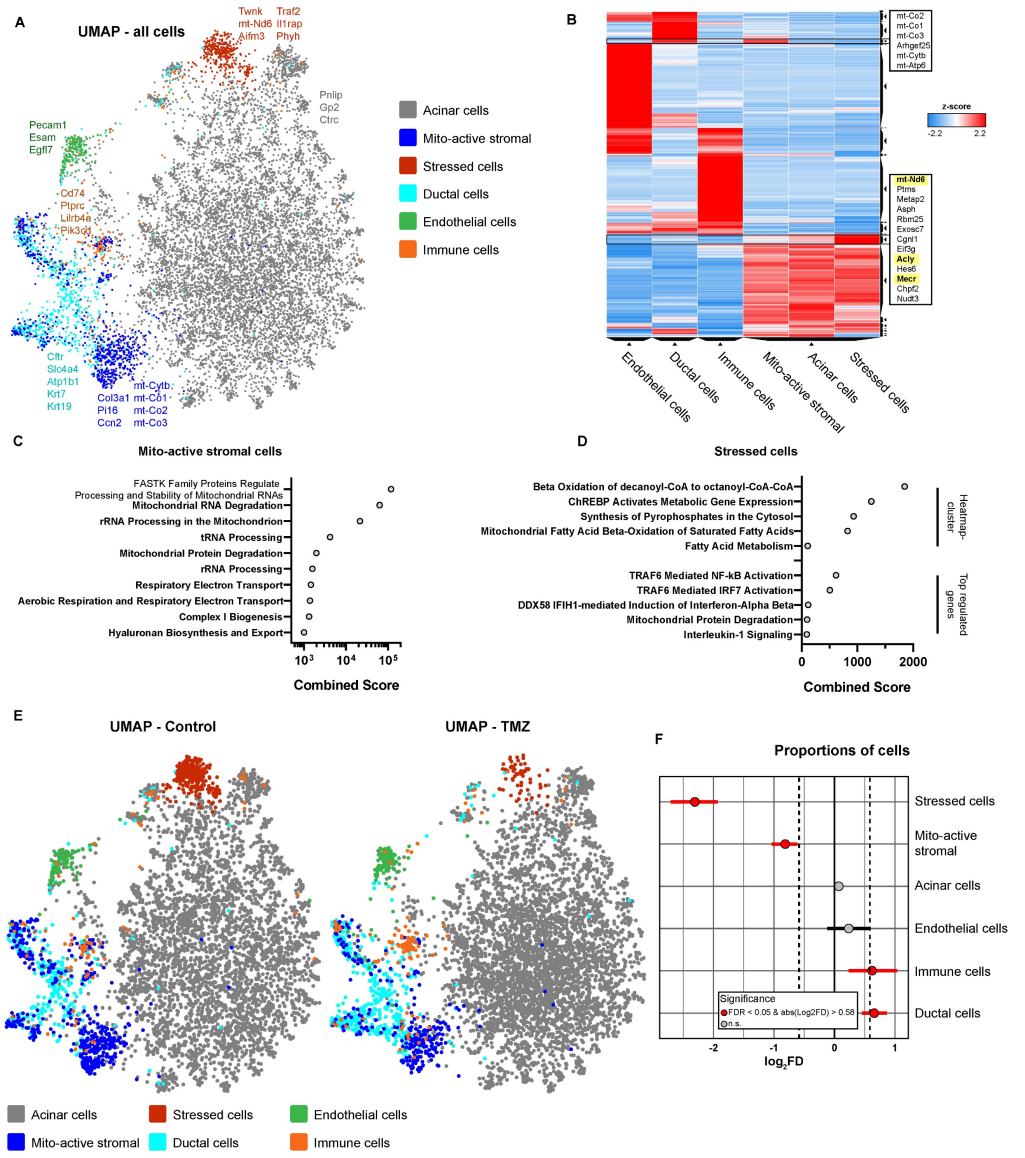
** Trimetazidine reduces mitochondrial and inflammatory stress-associated cell populations in dysglycemic pancreatic tissue.** (A) Uniform Manifold Approximation and Projection (UMAP) of scRNA-seq data from pre-diabetic mice, showing six major pancreatic cell clusters: acinar cells, mitochondrially active stromal cells, stressed cells, ductal cells, endothelial cells, and immune cells. (B) Clustergrammer heatmap illustrating cluster-specific gene expression profiles. Genes enriched in mitochondrially active stromal cells and stressed cells are highlighted. (C) Reactome pathway enrichment analysis (via Enrichr) of genes significantly upregulated in mitochondrially active stromal cells. (D) Reactome pathway enrichment analysis based on the heatmap-defined gene cluster and significantly upregulated genes in stressed cells. (E) UMAPs showing pancreatic cells from control and TMZ-treated pre-diabetic mice. (F) Statistical analysis of cluster-specific cell frequency changes in TMZ-treated animals.

To explore the organ-level effects of TMZ beyond its impact on T cells and diabetes onset, we performed single-cell RNA sequencing (scRNA-seq) on pancreatic tissue from dysglycemic NOD mice with blood glucose >150 mg/dL after one week of treatment (**Fig. 3A**). This approach enabled high-resolution profiling of early intervention alterations due to TMZ treatment in animals digressing from a pre-symptomatic stage to diabetes, providing insights into potential tissue-level effects of TMZ. Initial clustering of cells, followed by Enrichr-based cell identification 34-36, revealed a large acinar cell cluster, with exocrine pancreas signatures and high expression of digestive enzymes including *Pnlip*, *Gp2*, and *Ctrc*. Subclustering of the non-acinar compartment further resolved ductal cells, marked by expression of epithelial genes such as *Cftr*, *Slc4a4*, *Atp1b1*, *Krt7*, and *Krt19*, as well as endothelial cells expressing *Pecam1*, *Egfl7*, and *Esam*. Immune populations were annotated based on high expression of antigen-presenting cell markers (*Cd74*), pan-immune markers (*Ptprc/Cd45*), the myeloid marker (*Lilrb4a*), and the lymphoid marker (*Pik3cd*). We identified two transcriptionally distinct clusters within our pre-diabetic pancreas cells. Mitochondrially active stromal cells showed strong expression of mesenchymal markers (*Col3a1*, *Pi16*, *Ccn2*) and elevated mitochondrial genes (*mt-Cytb*, *mt-Co1, mt-Co2, mt-Co3*), consistent with enhanced metabolic activity. Stressed cells were characterized by the upregulation of multiple stress-associated genes, including mitochondrial (*Twnk, mt-Nd6*), oxidative and metabolic stress markers (*Aifm3, Phyh*), as well as pro-inflammatory signaling components (*Traf2, Il1rap*), indicating a transcriptional state of cellular stress and activation of stress-response pathways. UMAP visualization of all pancreatic cells across conditions is shown in **Fig. 3A**.

Differential gene expression analysis of mitochondrially active stromal cells, compared to all cells, shows broad upregulation of mitochondria-associated transcripts (**Fig. S6A**) as did an upregulated gene cluster in the Clustergrammer heatmap 38 (**Fig. 3B**). Finally, pathway analysis confirmed their high mitochondrial activity by finding several upregulated pathways related to mitochondrial RNA and protein processing, as well as aerobic respiration (**Fig. 3C**). In stressed cells, Clustergrammer revealed upregulated expression of *mt-Nd6, Acly,* and* Mecr* (**Fig. 3B**), which are related to fatty acid metabolism and especially FAO-related pathways (**Fig. 3D**). Differential gene expression analysis showed elevated levels of stress- and inflammation-related genes like *Traf2*, *IL1rap* or *Ifih1* (**Fig. S6B**). Pathway analysis confirmed enrichment for TRAF6 activation, IL-1 signaling, and mitochondrial protein degradation (**Fig. 3D**). Therefore, these cells were characterized by increased FAO pathways with pro-inflammatory and stress-related features.

Comparison of pre-diabetic control pancreatic cells with those treated *in vivo* with TMZ for one week, revealed pronounced changes in described cell populations. Most notably, stressed cells with high FAO were massively reduced in the TMZ treatment group (**Fig. 3E and S6C**). Further, also mitochondrially active stromal cells were decreased by TMZ treatment, showing both its inhibitory effect on mitochondria and FAO *in vivo* (**Fig. 3E**). Statistical analysis of cellular proportions 37 confirmed the reduction of stressed, FAO-high cells and mitochondrially active stromal cells (**Fig. 3F**). In contrast, immune and ductal cells were slightly increased. Notably, since the ductal cell cluster partially overlaps with mitochondrially active stromal cells, this increase may reflect a partial cellular shift from the latter (**Fig. 3F**).

Together, these findings demonstrate a decrease in stressed, pro-inflammatory cells, characterized by elevated FAO, and mitochondrially active stromal cells in pancreatic tissue from pre-diabetic mice treated with TMZ. This supports the *in vivo* inhibitory effects of TMZ on FAO and mitochondrial activity as shown in **Fig. 1**, consistent with its known anti-inflammatory properties 18. Overall, these data suggest improved pancreatic cellular health following one week of TMZ treatment in pre-diabetic mice, further reinforcing its tissue-protective potential.

### Prophylactic Trimetazidine treatment does not delay diabetes onset or alter islet immune cell infiltration in NOD mice

**Figure 4:**
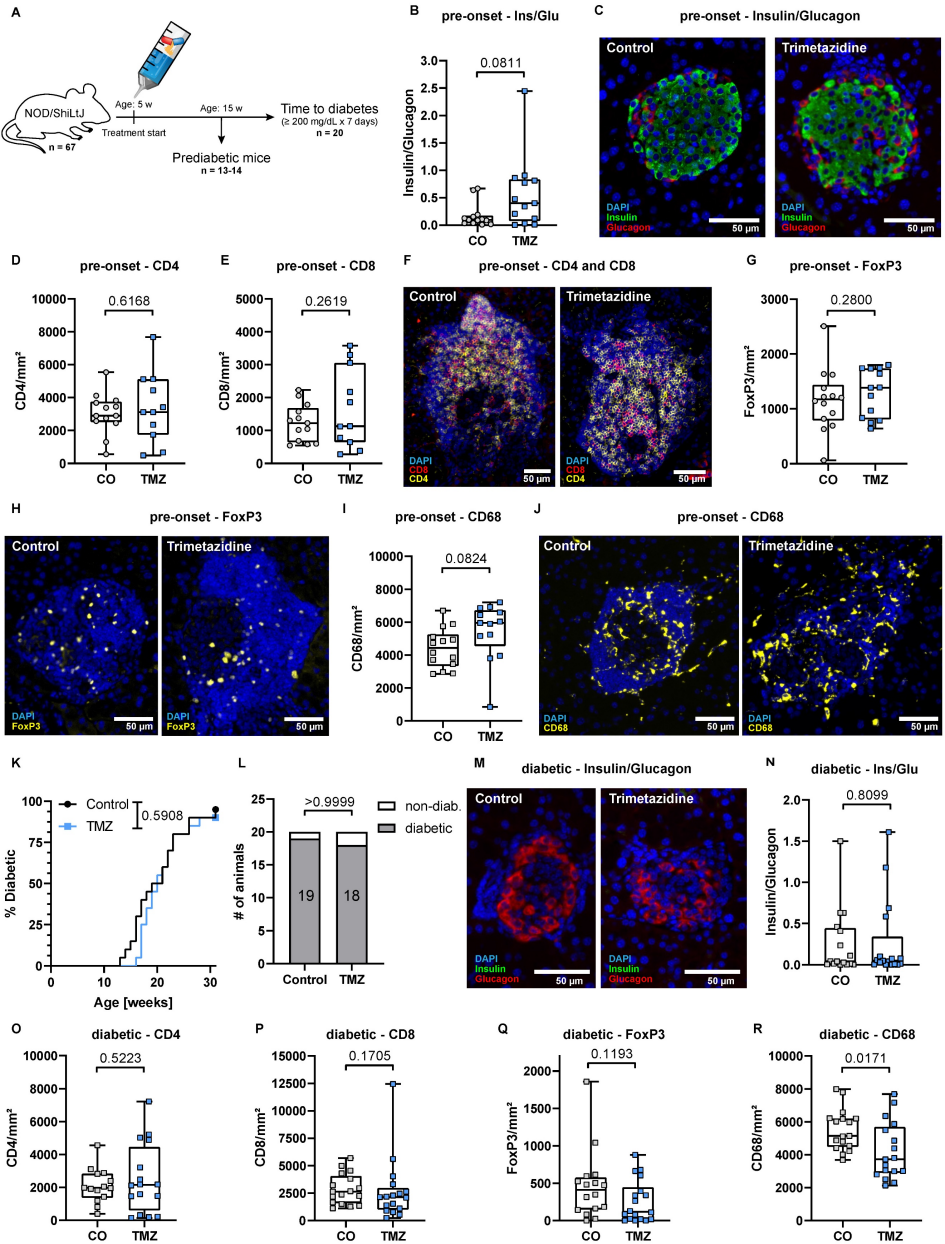
** Prophylactic TMZ treatment does not delay diabetes onset or alter islet immune infiltration in NOD mice.** (A) Experimental timeline: Female NOD/ShiLtJ mice received TMZ via drinking water from 5 weeks of age. A cohort of 13-14 mice was sacrificed at 15 weeks for analysis prior to diabetes onset, while 20 mice were monitored until either diabetes onset (defined as fasting blood glucose ≥200 mg/dL for one week) or 30 weeks of age, whichever comes first. (B) Quantification of β-cell (insulin) to α-cell (glucagon) ratios in pancreatic islets from pre-diabetes onset mice. Mean total islet area analyzed per mouse: 175,385 µm². n= 13-14. (C) Representative images showing insulin^+^ β-cells and glucagon^+^ α-cells in islets from pre-diabetes onset mice. (D) Quantification of CD4^+^ and (E) CD8^+^ T cell infiltration in islets from pre-diabetes onset mice. Mean total islet area analyzed per mouse: 112,745 µm². n= 11-13. (F) Representative images of CD4^+^ and CD8^+^ T cell infiltration in islets from pre-diabetes onset mice. (G) Quantification of FoxP3^+^ regulatory T cells in islets from pre-diabetes onset mice. Mean total islet area analyzed per mouse: 175,385 µm². n= 13-14. (H) Representative images of FoxP3^+^ regulatory T cell infiltration in islets from pre-diabetes onset mice. (I) Quantification of CD68^+^ macrophage in islets from pre-diabetes onset mice. Mean total islet area analyzed per mouse: 202,532 µm². n= 13-14. (J) Representative images of CD68^+^ macrophage infiltration in islets from pre-diabetes onset mice. (K) Kaplan-Meier analysis of diabetes onset in TMZ-treated and control mice. P-value calculated with the Gehan-Breslow-Wilcoxon test. n= 20. (L) Final diabetes incidence in both groups. n=20. Statistical comparison was performed using Fisher's exact test. (M) Representative images of insulin and glucagon staining in diabetic pancreases. (N) Quantification of β-cell (insulin) to α-cell (glucagon) ratios in diabetic islets. Mean total islet area analyzed per mouse: 88,582 µm². n=16-17. (O) Quantification of CD4^+^ T cell infiltration in diabetic islets. Mean total islet area analyzed per mouse: 77,100 µm². n= 14-16. (P) Quantification of CD8^+^ T cell infiltration in diabetic islets. Mean total islet area analyzed per mouse: 77,100 µm². n= 17. (Q) Quantification of FoxP3^+^ T cell infiltration in diabetic islets. Mean total islet area analyzed per mouse: 88,582 µm². n= 16-18. (R) Quantification of CD68^+^ macrophages in diabetic islets. Mean total islet area analyzed per mouse: 130,986 µm². n= 17. CO: Control; TMZ: Trimetazidine. Unpaired t-test or Mann-Whitney test were used for statistical analysis.

T1D is preceded by early insulitis and autoimmune activation, and genetic markers such as the HLA-DQ8 haplotype enable reliable risk prediction 49. This has paved the way for targeted prophylaxis, as demonstrated by the preventive use of the anti-CD3 antibody teplizumab 3. To model prophylaxis with TMZ, we treated female NOD/ShiLtJ mice starting at 5 weeks of age, a time point corresponding to the onset of insulitis in this strain 28. 14 control and 13 TMZ-treated mice were sacrificed at 15 weeks to assess treatment effects before diabetes onset, while 20 mice per group were monitored until diabetes onset or until reaching 30 weeks of age (**Fig. 4A**).

In mice before diabetes onset, the β- to α-cell ratio remained unchanged following 10 weeks TMZ treatment (**Fig. 4B**) and insulin-producing β-cells were still readily detectable (**Fig. 4C**). In contrast to acute TMZ intervention, prophylaxis did not affect infiltration of CD4^+^ or CD8^+^ T cells (**Fig. 4D-F**). Similarly, the abundance of FoxP3^+^ regulatory T cells (**Fig. 4G-H**) and islet-associated macrophages (**Fig. 4I-J**) was unaltered. Circulating immune populations also remained largely unaffected, with only a slight increase in Ly6G^+^ neutrophils and a minor decrease in Ly6C^low^ monocytes (**Fig. S7A-F**). Circulating CD4^+^ and CD8^+^ T cells, including their respective subsets, were unchanged by TMZ treatment (**Fig. S7G-H**). Prophylactic TMZ treatment did not delay diabetes onset. Kaplan-Meier analysis showed no significant difference in the time to diabetes onset between the two groups (**Fig. 4K**). 19 out of 20 control mice and 18 out of 20 TMZ-treated mice eventually developed diabetes (**Fig. 4L**).

In diabetic mice, β-cells were markedly reduced and scarcely detectable in both control animals and TMZ treated mice (**Fig. 4M**). The β- to α-cell ratio remained unchanged by TMZ treatment (**Fig. 4N**). CD4^+^, CD8^+^, and Treg infiltration was unchanged (**Fig. 4O-Q**), with only islet-associated macrophages reduced in TMZ treated animals (**Fig. 4R**). Circulating immune cells and T cell subsets remained unaffected by treatment (**Fig. S8A-H**).

These findings suggest that while TMZ exerts acute immunomodulatory and cytoprotective effects, its long-term prophylactic administration does not prevent disease progression in the NOD model.

### Trimetazidine transiently impairs CD8^+^ T cell differentiation *in vitro*

**Figure 5:**
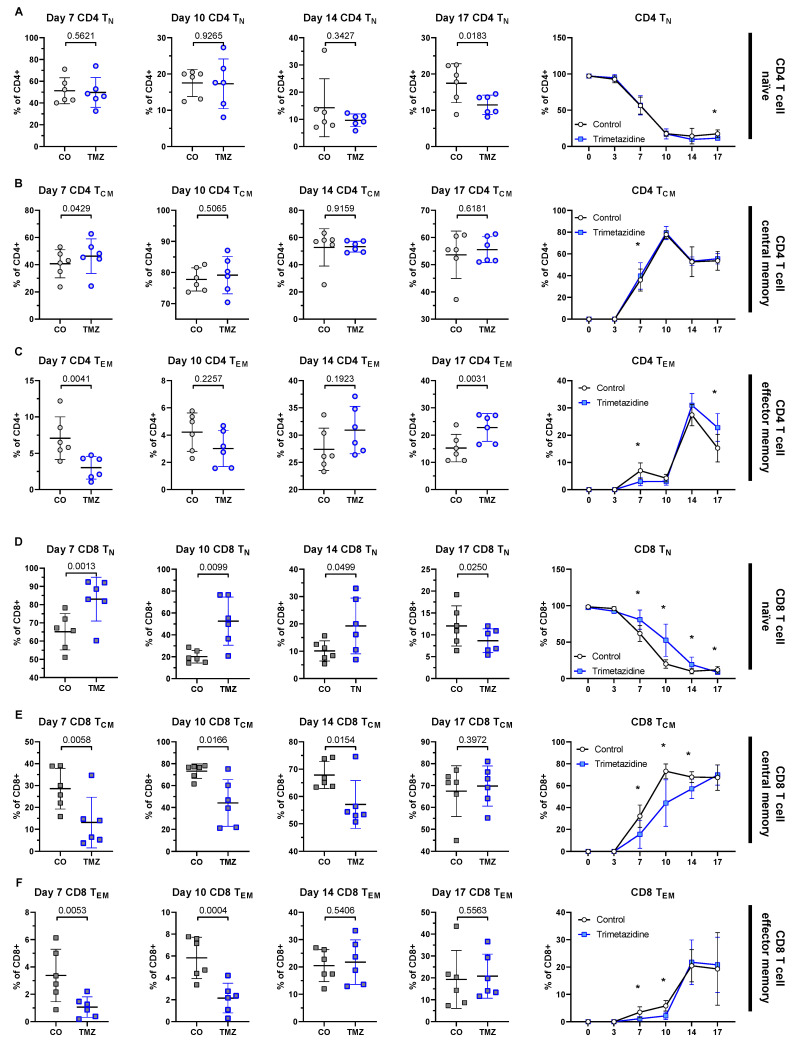
** Trimetazidine modulates CD4^+^ and CD8^+^ T cell differentiation *in vitro*.** Naïve human CD4^+^ and CD8^+^ T cells were cultured under stimulatory conditions with or without TMZ for 17 days. Surface expression of CCR7 and CD45RA was used to define T cell subsets by flow cytometry: naïve (T_N_; CCR7^+^ CD45RA^+^), central memory (T_CM_; CCR7^+^CD45RA^-^), and effector memory (T_EM_; CCR7^-^ CD45RA^-^). (A) Percentage of CD4^+^ T_N_ cells across days 7-17 and time-course analysis from day 0 to 17. (B) CD4^+^ T_CM_ cells across days 7-17 and time-course analysis. (C) CD4^+^ T_EM_ cells across days 7-17 and time-course analysis. (D) CD8^+^ T_N_ cells across days 7-17 and time-course analysis. (E) CD8^+^ T_CM_ cells across days 7-17 and time-course analysis. (F) CD8^+^ T_EM_ cells across days 7-17 and time-course analysis. Data represent n = 6 donors. Paired t-test was used for statistical analysis. * indicates p < 0.05. CO: Control; TMZ: Trimetazidine.

While acute TMZ intervention delayed CD4^+^ and CD8^+^ T cell infiltration into pancreatic islets, this effect was not maintained in mice after extended treatment, nor in mice that received TMZ prophylactically. To understand the underlying mechanisms, we assessed the differentiation of naïve CD4^+^ and CD8^+^ T cells (T_N_) *in vitro* in the presence or absence of TMZ for up to 17 days by flow cytometry (**Fig. S9A**). Culturing naïve CD4^+^ T cells with TMZ over 17 days exerted only modest effects. CD4^+^ T_N_ gradually declined over the culture period (**Fig. 5A**) and CD4^+^ central memory T cells (T_CM_) were transiently increased by TMZ at day 7 but showed no lasting change (**Fig. 5B**). CD4^+^ effector memory T cells (T_EM_) were the most affected subset, with a significant reduction at day 7 and a compensatory increase by day 17 (**Fig. 5C**). CD4^+^ terminally differentiated effector memory T cells re-expressing CD45RA (T_EMRA_) remained unchanged (**Fig. S9B**). In contrast, CD8^+^ T cells, which play a central role in β-cell destruction during T1D, were more consistently affected by the drug intervention. TMZ treatment led to a sustained retention of CD8^+^ T_N_ cells from day 7 to day 17 (**Fig. 5D**). This was accompanied by a marked reduction in CD8^+^ T_CM_ cells between day 7 and day 14 (**Fig. 5E**) and in CD8^+^ T_EM_ cells from day 7 to 10 (**Fig. 5F**). The T_EMRA_ fraction in CD8^+^ T cells remained low in both groups and was unaffected by treatment (**Fig. S9C**).

These findings suggest that TMZ exerts a transient inhibitory effect on CD8^+^ T cell differentiation, particularly evident between days 7 and 14 of stimulation. This early blockade is compensated at day 17, indicating a dynamic adaptation of CD8^+^ T cell fate under metabolic restriction. This transient modulation may explain why the protective effects of TMZ observed in acute settings are not sustained during long-term treatment.

### CD8^+^ T cells compensate trimetazidine-induced metabolic modulation by upregulating CPT1A

TMZ inhibits 3-ketoacyl-CoA-thiolase (HADHB), the final enzyme of mitochondrial β-oxidation, thereby reducing FAO 15, 50 (**Fig. 1E**). CD8^+^ T_CM_ were the most affected populations in our *in vitro* differentiation assays (**Fig. 5E**), reflecting that memory CD8^+^ T cells are particularly dependent on FAO during their development 51. To obtain subset-specific data on cell metabolism, we employed a previously published, metabolic flow cytometry panel 52 that allowed us to quantify the abundance of key metabolic proteins within each CD8^+^ T cell subset and to capture adaptive metabolic changes under TMZ treatment *in vitro*. We focused on four critical metabolic regulators: CPT1A, the rate-limiting enzyme of FAO 53, 54, GLUT1 (glucose transporter 1), the main glucose transporter 55, PRDX2 (peroxiredoxin-2), an antioxidant enzyme that counters oxidative stress 56-58, and IDH2 (NADP^+^-dependent isocitrate dehydrogenase 2), a TCA cycle enzyme 59.

**Figure 6:**
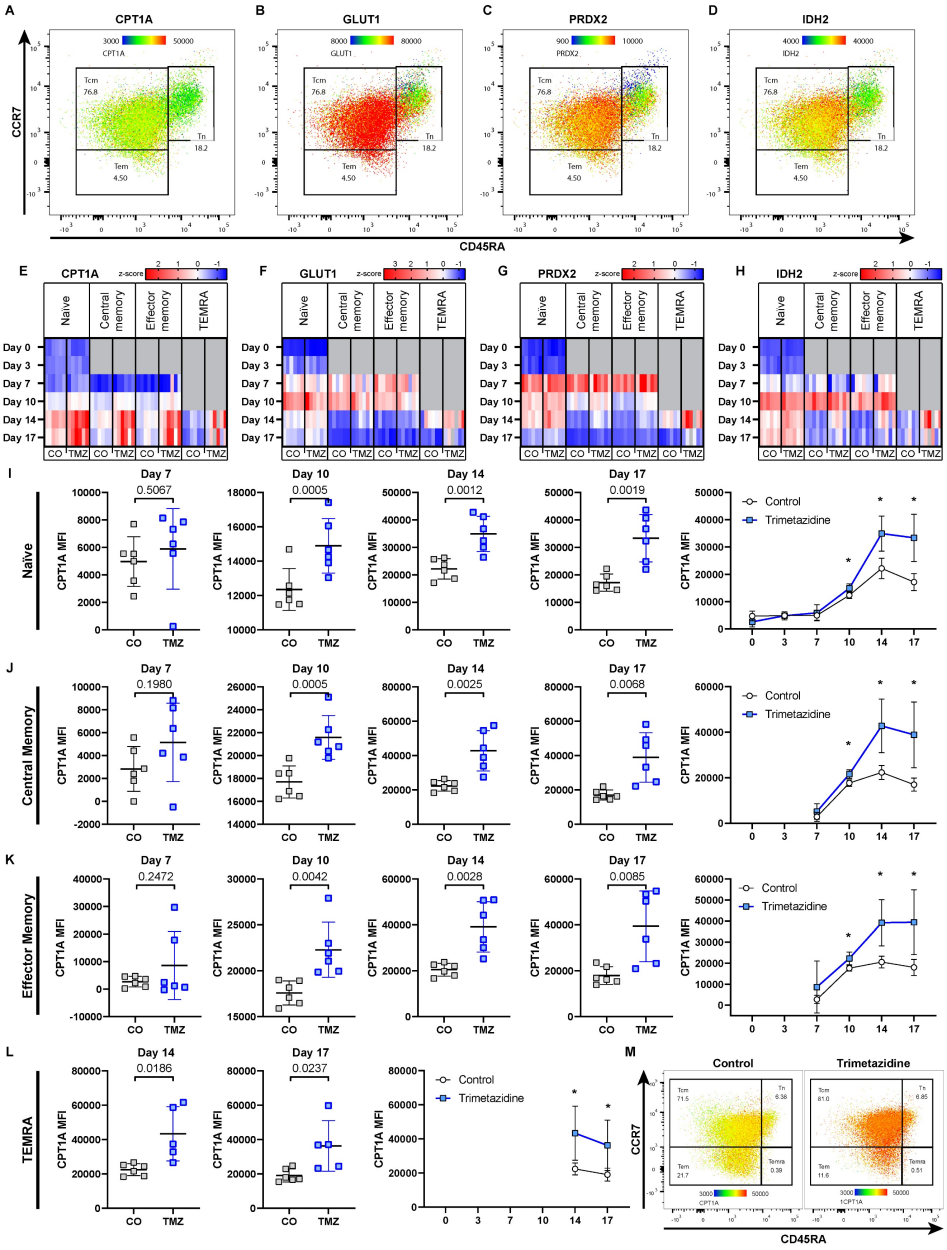
** Metabolic compensation of CD8^+^ T cells under Trimetazidine treatment.** Naïve human CD8^+^ T cells were cultured under stimulatory conditions with or without TMZ for 17 days. Surface expression of CCR7 and CD45RA was used to define T cell subsets by flow cytometry: naïve (T_N_; CCR7^+^CD45RA^+^), central memory (T_CM_; CCR7^+^CD45RA^-^), effector memory (T_EM_; CCR7^-^CD45RA^-^), and T_EMRA_ (CCR7^-^CD45RA^+^). (A-D) Representative heatmap-style dot plot from intracellular flow cytometry showing expression of CPT1A (carnitine-palmitoyl-transferase-1A) (A), GLUT1 (glucose transporter 1) (B), PRDX2 (peroxiredoxin-2) (C), and IDH2 (NADP^+^-dependent isocitrate dehydrogenase 2) (D) in control CD8^+^ T cell subsets (T_N_, T_CM_, T_EM_) at day 10. Dot color shows mean fluorescence intensity, thus indicates protein abundance. (E-H) Heatmaps of z-scored expression levels for CPT1A (E), GLUT1 (F), PRDX2 (G), and IDH2 (H) across CD8^+^ T cell subsets and all time points (day 0, 3, 7, 10, 14, 17) under control and TMZ conditions. (I-L) Quantification of CPT1A expression over time in CD8^+^ T_N_ (I), T_CM_ (J), T_EM_ (K), and T_EMRA_ (L) subsets. (M) Heatmap-style dot plot showing CPT1A expression across all subsets at day 17, highlighting elevated expression in all TMZ-treated populations. Data represent n = 5-6 donors. Paired t-test was used for statistical analysis. * indicates p < 0.05. CO: Control; TMZ: Trimetazidine.

These markers revealed subset-specific metabolic profiles in T_N_, T_CM_, and T_EM_ CD8^+^ T cells (**Fig. 6A-D**), which changed dynamically over time and in response to TMZ treatment (**Fig. 6E-H**). In control conditions, with the first appearance of T_CM_ and T_EM_ at day 7, we observed an initial metabolic shift, marked by increased levels of GLUT1, PRDX2, and IDH2 across all subsets compared to day 3. This pattern suggested an increased energy demand via glycolysis (GLUT1) and aerobic respiration (IDH2), as well as reduction of ROS generation (PRDX2). By day 10, β-oxidation increased (CPT1A), along with further elevations of GLUT1 and IDH2, indicating that the cells increasingly relied on glycolysis, the TCA cycle, and β-oxidation to meet rising energy demands. By day 14, levels of GLUT1, PRDX2, and IDH2 declined in T_CM_ and T_EM_, while CPT1A remained elevated, reflecting a shift toward FAO dependency in these memory subsets.

Within the treatment group, of all markers, CPT1A showed the most pronounced TMZ-induced changes, with increased expression starting at day 10 in all CD8^+^ T cell subsets. TMZ-treated CD8^+^ T cells showed sustained CPT1A upregulation across all populations, T_N_ (**Fig. 6I**), T_CM_ (**Fig. 6J**), T_EM_ (**Fig. 6K**), and T_EMRA_ (**Fig. 6L**), with a heatmap-style dot plot confirming this increase at day 17, suggesting compensatory FAO entry activation in response to downstream enzymatic blockade (**Fig. 6M**). GLUT1 was transiently reduced until day 10, then rebounded by day 17 (**Fig. S10A**). PRDX2 was upregulated from day 10 onward, consistent with increased oxidative stress (**Fig. S10B**). IDH2 increased in T_EM_ by day 10 and in other subsets by day 14 (**Fig. S10C**). These adaptations were also reflected at the organ level. To assess this, we analyzed pancreatic tissue from pre-onset and diabetic mice by qPCR, normalizing expression to the respective control group within each stage. In pre-onset mice, *Cpt1a*, *Idh2*, and *Prdx2* mRNA levels were reduced in TMZ-treated animals compared to controls. In contrast, diabetic mice showed no difference between treatment groups, showing unified expression profiles. *Slc2a1* (*Glut1*) remained unchanged in both conditions (**Fig. S11A-B**).

Given that TMZ targets HADHB, we were interested if these dynamics would be altered *in vitro* and *in vivo* similar to CPT1. We found increased levels of HADHB at day 7 in naïve CD8 T cells indicating an early upregulation of the drug target possibly supporting the observed escape from drug treatment (**Fig. S12A**). At day 17, where the T cell differentiation effect was recovered, HADHB was no longer increased (**Figure S12B**). Reanalysing our single cell RNA seq data for HADHB and CPT1A we found that 7 days after TMZ treatment, immune cells showed a strong upregulation of HADHB with mito-active stromal cells demonstrating increased CPT1A levels (**Figure S12C**).

In contrast, CD4^+^ T cells exhibited minimal metabolic changes in response to TMZ, with only the T_N_ subset showing slight alterations, while the other subsets remained largely stable (**Fig. S13A-H**). This aligns with our previous observations showing limited effects of TMZ on CD4^+^ T cell differentiation (**Fig. 5A-C**).

Taken together, these results indicate that CD8^+^ T cells respond to TMZ-induced FAO inhibition by upregulating CPT1A and HADHB, reflecting a metabolic adaptation attempting to compensate for impaired fatty acid utilization. This compensatory mechanism may explain the short-lived efficacy of TMZ in modulating T cell function *in vivo*.

## Discussion

Current strategies to delay type 1 diabetes (T1D) onset remain limited, with teplizumab being the only approved therapy delaying the onset of disease by several years 60. The mechanism of teplizumab involves partial signaling via CD3 with subsequent deactivation inducing T cell exhaustion of especially CD8^+^ T cells and restoring self-tolerance 61. Given that especially CD8^+^ T cells are specifically associated with FAO 6, 51, 52 we tested the effect of the FAO inhibitor trimetazidine in the setting of T1D.

Inhibiting FAO during the initial activation of human T cells with TMZ led to reduced activation and proliferation. Especially CD28 signaling was previously reported to activate and prime mitochondria and promote FAO 62. We therefore suggest that within the initial activation step, TMZ treatment reduces overall cell metabolism thereby preventing T cell activation. When analyzing the differentiation potential of naïve T cells during TMZ treatment over a total of 17 days, we found only minimal differences in resulting CD4^+^ T cell populations but a significant alteration in differentiation patterns for CD8^+^ T cells. This probably reflects that CD4^+^ T cells rely predominantly on glycolysis. Within CD8^+^ T cells FAO does not only provide necessary energy but also drives cell cycle progression 63. This is reflected in our long term data set with a delayed differentiation of naïve CD8^+^ T cells over time. Interestingly, after 17 days of differentiation, TMZ inhibition is circumvented, leading to similar amounts of central and effector memory CD8^+^ T cells.

T cells have been already reported to be able to escape drug induced inhibition. Human Th17 cells were demonstrated to be resistant to glucocorticoid treatment due to presence of MDR1 reducing the effect of intracellular glucocorticoid exposure 64. Under strong T cell receptor activation, T cells were able to circumvent rapamycin and its inhibitory effect on proliferation via upregulation of bcl-xl and downregulation of p27^kip1^ 65. We therefore tested if CD8^+^ T cells actively rearrange their metabolism to counteract TMZ treatment. Rather than an obvious switch away from mitochondrial metabolism, the dominant measurable response to escape TMZ induced inhibition was CPT1A and 3-ketacyl-CoA thiolase (HADHB) upregulation with 3-ketacyl-CoA dynamics appearing similar in *in vitro* and *in vivo* experiments. We further suggest that the observed loss of function of TMZ in our experimental setup might be additionally in part due to its reversible competitive nature as suggested by Lopaschuk et al 50 allowing for substrate processing when critical amounts are reached. Our data thereby demonstrate the necessity of FAO in CD8^+^ T cells with upregulation of FAO pathway components to allow for respective substrate use.

Our *in vivo* data confirm our *in vitro* observations. In the NOD mouse model of spontaneous T1D development, treatment with TMZ delayed the onset of disease. One week after TMZ initiation, the pancreas of treated mice displayed reduced metabolic stress and diminished T cell infiltration. This time point reflects peak autoimmune activity and the capability of TMZ to inhibit this initial T cell activation. However, we already observed the compensatory upregulation of HADHB in immune cells at this stage similar to our observations in the *in vitro* model system. Consequently, with a significant delay we also observed diabetes onset in TMZ treated animals. With established disease, we did not observe alterations in T cell numbers within the pancreas between the control and treatment group. T cell numbers were overall reduced in pancreatic tissue during established disease. We suggest that this reflects cell dynamics reported for diabetes models as previous reports already demonstrated increased T cell infiltration during insulitis induced β-cell destruction with a decrease at later stages probably due to antigen loss. Reddy et al reported that islets that stained negative for insulin were also negative for peri-islet T cells four weeks after diabetes onset 66, 67. Overall, we further propose that the observed lack of efficacy of TMZ in a prophylactic model is a reflection of metabolic adaptation to TMZ induced FAO inhibition.

We further suggest that the predominant effect of TMZ in delaying T1D onset is caused by a transient inhibition of T cells. Bulk RNA sequencing of human pancreatic β-cells treated with TMZ revealed only modest direct transcriptional changes induced by TMZ, without enrichment of metabolic or stress-related gene sets. Among the few enriched gene sets we observed was heme scavenging, involving low-density lipoprotein receptor-related protein 1 (LRP1), which has been linked to β-cell protection in type 2 diabetes 68.

Previously, we described that TMZ reduces the proinflammatory potential in macrophages in atherosclerotic lesions 18. In T1D, macrophages initiate lymphocyte recruitment to pancreatic islets, release pro-inflammatory cytokines and present auto-antigens during β-cell destruction 69. We found in TMZ-treated animals a significant reduction of pancreatic macrophages compared to control animals specifically in the diabetic group. Nonetheless, this macrophage reduction was not sufficient to alter T1D progression.

Building on our findings, future work could broaden the mechanistic framework by considering that TMZ may exert additional, cell type-specific effects beyond partial FAO inhibition, including modulation of intracellular signaling nodes such as STAT3 and NF-κB 70. In this context, recent evidence that canonical and non-canonical STAT3/NF-κB dimer composition can interact to regulate pineal and extra-pineal melatonin synthesis raises the possibility that TMZ may also influence the endogenous mitochondrial melatonergic pathway in pancreatic β cells and CD8⁺ T cells 71. This is of particular interest because melatonin has been linked to β-cell protection in the inflammatory milieu relevant to T1D 72, 73.

In conclusion, our data demonstrate that TMZ alters T cell metabolism and differentiation by inhibiting FAO. However, CD8^+^ T cells adapt through intrinsic metabolic reprogramming leading only to a transient effect.

## Conclusions

Type 1 diabetes remains a disease where immunotherapy can delay but rarely prevent progression, as exemplified by teplizumab 74. Our study adds an important mechanistic layer to this challenge demonstrating that pathogenic CD8⁺ T cells exhibit metabolic flexibility, adapting to FAO inhibition by upregulating CPT1A and HADHB, which restores their effector function. By using the clinically approved FAO modulator trimetazidine as a tool, we demonstrate both the potential and the limitations of metabolic interventions in autoimmune diabetes. These findings highlight that the barrier to long-term efficacy is not the absence of drug activity, but rather the adaptive capacity of autoreactive T cells. Understanding and overcoming this metabolic resilience will be critical to move beyond transient delay and towards durable prevention of T1D progression.

## Supplementary Material

Supplementary figures.

## Figures and Tables

**Table 1:** Primer sequences used for quantitative PCR analysis.

Primer Target	Primer Sequence
18S-FWD	CTTAGAGGGACAAGTGGCG
18S-REV	ACGCTGAGCCAGTCAGTGTA
CXCL10-FWD:	GCATTCAAGGAGTACCTCTCTC
CXCL10-REV:	CAGACATCTCTTCTCACCTTC
HLA-B-FWD:	CTGGAGAAGAGCAGAGATACAC
HLA-B-REV:	TTTCCACCTGAACTCTTCCTC
Cpt1a-FDW:	AGGTTCAAGCTGTTCAAGATAG
Cpt1a-REV:	TCAGACAGTACCTCCTTCAG
Idh2-FWD:	CATTACCGAGAACACCAGAAG
Idh2-REV:	CGTCTGTGCAAACCTGATAA
Prdx2-FWD:	TTCGGACTACAGAGGGAAG
Prdx2-REV:	AACTGAGAGTCCACAGACA
Slc2a1-FWD:	CTTCATCATCGGTGTGTACTG
Slc2a1-REV:	CCAAACACCTGGGCAATAA

**Table 2:** Antibodies used for flow cytometric analysis of murine and human samples

Murine blood leukocyte phenotyping:				
**Target**	**Fluorophore**	**Clone**	**Dilution**	**Company**
CD45	BV650	30-F11	1:200	Biolegend
CD41	PerCP/Cy5.5	MWReg	1:200	Biolegend
Ly6G	APC	1A8	1:200	Biolegend
CD115	PE	AFS98	1:200	Biolegend
Ly6C	BV605	HK1.4	1:200	Biolegend
CD3e	FITC	145-2C11	1:200	Biolegend
B220	BV421	RA3-6B2	1:200	Biolegend
Murine T cell phenotyping in blood and pancreatic lymph nodes:				
**Target**	**Fluorophore**	**Clone**	**Dilution**	**Company**
CD3	APC/Cy7	17A2	1:100	Biolegend
CD4	PerCP	RM4-5	1:100	Biolegend
CD8	BV605	53-6.7	1:50	Biolegend
CD25	PE/Cy7	3C7	1:100	Biolegend
CD44	PE	IM7	1:100	Biolegend
CD62L	APC	MEL-14	1:100	Biolegend
*Intracellular:*				
FoxP3	BV421	FJK-16s	1:50	Thermo Fisher
Cultured human T cells				
**Target**	**Fluorophore**	**Clone**	**Dilution**	**Company**
CD8a	BV785	RPA-T8	1:100	Biolegend
CCR7 (CD197)	PE/Cy7	G043H7	1:100	Biolegend
CD4	PerCP/Cy5.5	RPA-T4	1:100	Biolegend
CD45RA	APC/Cy7	HI100	1:100	Biolegend
*Intracellular:*				
CPT1A	V450	8F6AE9	1:100	BD
IDH2	PE	EPR7577	1:200	Abcam
GLUT1	Alexa Fluor 488	EPR3915	1:200	BD
PRDX2	Alexa Fluor 647	EPR5154	1:200	BD

**Table 3:** Primary and secondary antibodies used for immunofluorescence staining of pancreatic sections.

Target	Concentration	Clone	Catalog #	Company	Antigen Retrieval
Insulin	0.0556 µg/mL	RM1019	ab282459	Abcam	Citrate
Glucagon	0.1 µg/mL	ICALS	14-9743-82	Thermo Fisher	Citrate
CD4	5 µg/mL	BLR167J	MA5-44519	Thermo Fisher	Tris-EDTA
CD8a	5 µg/mL	4SM16	14-0195-82	Thermo Fisher	Citrate
FoxP3	5 µg/mL	FJK-16s	14-5773-82	Thermo Fisher	Citrate
CD68	4.37 µg/mL	EPR23917-164	ab283654	Abcam	Tris-EDTA
anti-mouse IgG DyLight 488	2.5 µg/mL	-	SA5-10166)	Thermo Fisher	-
anti-mouse IgG Alexa Fluor 555	10 µg/mL	-	A31570)	Thermo Fisher	-
anti-rabbit IgG Dylight 488	2.5 µg/mL	-	SA5-10038)	Thermo Fisher	-
anti-rabbit IgG Dylight 650	2.5 µg/mL	-	SA5-10041)	Thermo Fisher	-
anti-rat IgG Dylight 550	2.5 µg/mL	-	SA5-10027)	Thermo Fisher	-
anti-rat IgG Dylight 650	2.5 µg/mL	-	SA5-10029)	Thermo Fisher	-

## Data Availability

Data is available from the corresponding author upon reasonable request. The RNA-seq data is available under GEO accession: GSE301646.

## Abbreviations

ATPCI: A Study to Assess the Efficacy and Safety of Trimetazidine in patients with angina pectoris treated by Percutaneous Coronary Intervention
CD: Cluster of Differentiation
CPT1A: Carnitine Palmitoyltransferase 1A
DAPI: 4′,6-Diamidino-2-Phenylindole
ELISA: Enzyme-Linked Immunosorbent Assay
FAO: Fatty Acid Oxidation
FBS: Fetal Bovine Serum
GSIS: Glucose-Stimulated Insulin Secretion
GSEA: Gene Set Enrichment Analysis
HADHB: 3-ketacyl-CoA thiolase
IFN: Interferon
IL: Interleukin
LRP1: Low-Density Lipoprotein Receptor-Related Protein 1
NOD: Non-Obese Diabetic (mouse)
OXPHOS: Oxidative Phosphorylation
PBS: Phosphate-Buffered Saline
PLN: Pancreatic Lymph Node
PRDX2: Peroxiredoxin 2
qPCR: Quantitative Polymerase Chain Reaction
RNA-seq: RNA Sequencing
ROS: Reactive Oxygen Species
RPMI: Roswell Park Memorial Institute Medium
scRNA-seq: Single-Cell RNA Sequencing
T1D: Type 1 Diabetes
T2D: Type 2 Diabetes
TCA: Tricarboxylic Acid (cycle)
TCM: Central Memory T Cell
TEM: Effector Memory T Cell
TEMRA: Terminally Differentiated Effector Memory T Cell re-expressing CD45RA
TMZ: Trimetazidine
TN: Naïve T Cell
Treg: Regulatory T Cell
UMAP: Uniform Manifold Approximation and Projection
