# Supplementary material for: Metabolic Adaptation of CD8⁺ T Cells Limits the Efficacy of Fatty Acid Oxidation Inhibition in Type 1 Diabetes
Source: Int J Biol Sci. 2026 Jan 30;22(5):2196–214. doi: 10.7150/ijbs.125649 (PMC12965062; doi:10.7150/ijbs.125649)
Supplement: Supplementary file 1 — Supplementary figures. [file ijbsv22p2196s1.pdf]

Figure S1

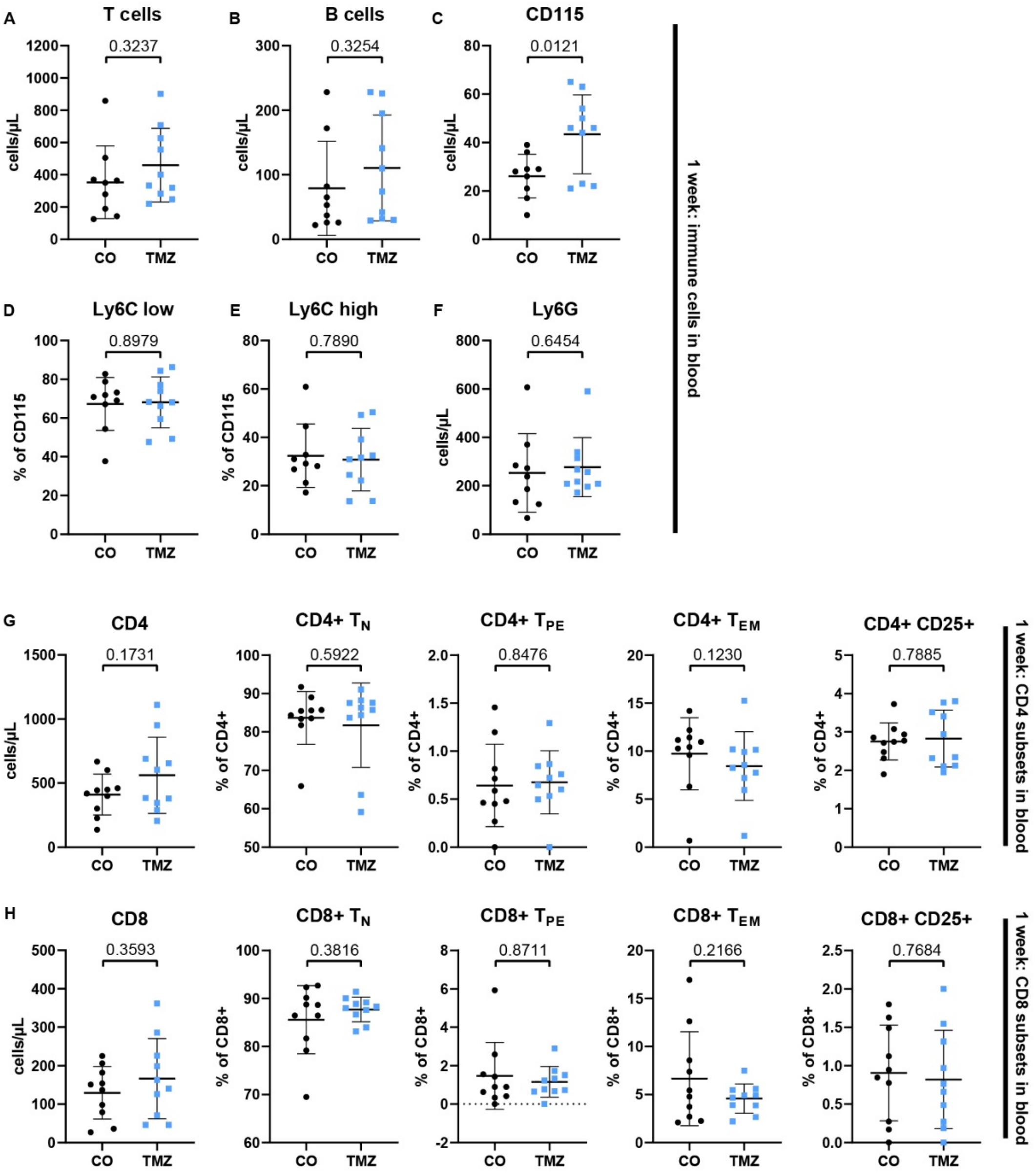

Figure S1. Flow cytometric analysis of circulating immune cell populations in NOD mice treated with Trimetazidine (TMZ)

Circulating immune cells after one week of TMZ treatment:
(A) CD3^{+} T cells, (B) B220^{+} B cells, (C) CD115^{+} monocytes, (D) Percentage of Ly6Cl^{ow} CD115^{+} monocytes, (E) Percentage of Ly6C^{high} CD115^{+} monocytes, (F) Ly6G^{+} neutrophils.
(G) CD4^{+} T cells and their subsets: T_{N}, T_{PE}, T_{EM}, and CD25^{+} cells, (H) CD8^{+} T cells and their subsets: T_{N}, T_{PE}, T_{EM}, and CD25^{+} cells.

(A-F) n= 9-10, (G,H) n= 10. Unpaired t-test or Mann-Whitney test were used for statistical analysis. CO: Control; TMZ: Trimetazidine; T_{N}: naïve T cells; T_{PE}: pre-effector T cells; T_{EM}: effector memory T cells

Figure S2

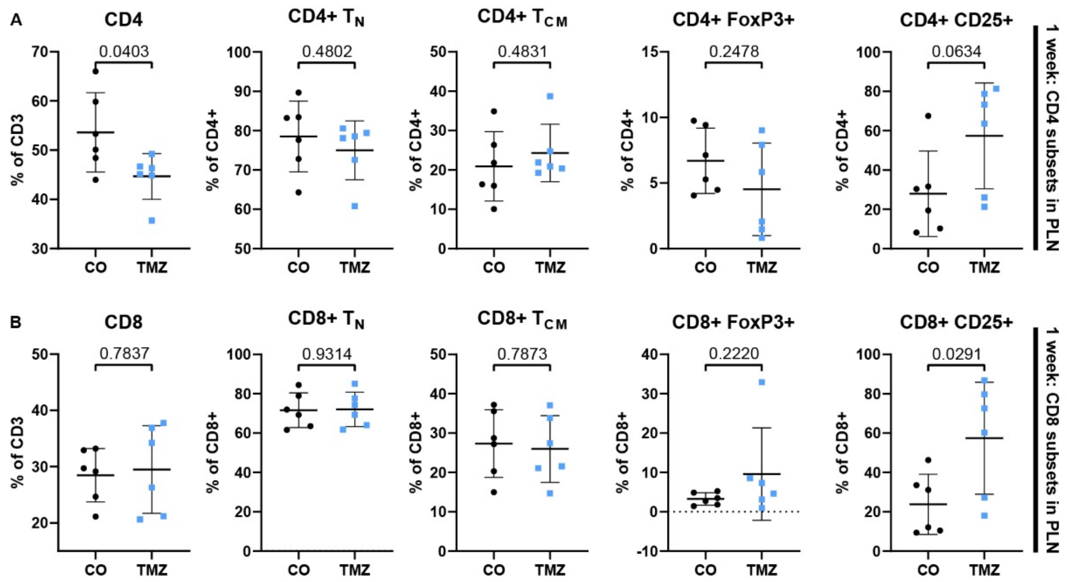

Figure S2. Flow cytometric analysis of lymphoid immune cell populations in NOD mice treated with Trimetazidine (TMZ)

Pancreatic lymph nodes were analyzed by flow cytometry to assess T cells subsets after one week of TMZ treatment:

(A) $CD4^+$ T cells and their subsets: T_N, T_{CM}, $FoxP3^+$ regulatory T cells, and $CD25^+$ T cells.
(B) $CD8^+$ T cells and their subsets: T_N, T_{CM}, $FoxP3^+$ regulatory T cells, and $CD25^+$ T cell.

n= 6. Unpaired t-test was used for statistical analysis.
CO: Control; TMZ: Trimetazidine; T_N: naïve T cells; T_{CM}: central memory T cells

Figure S3

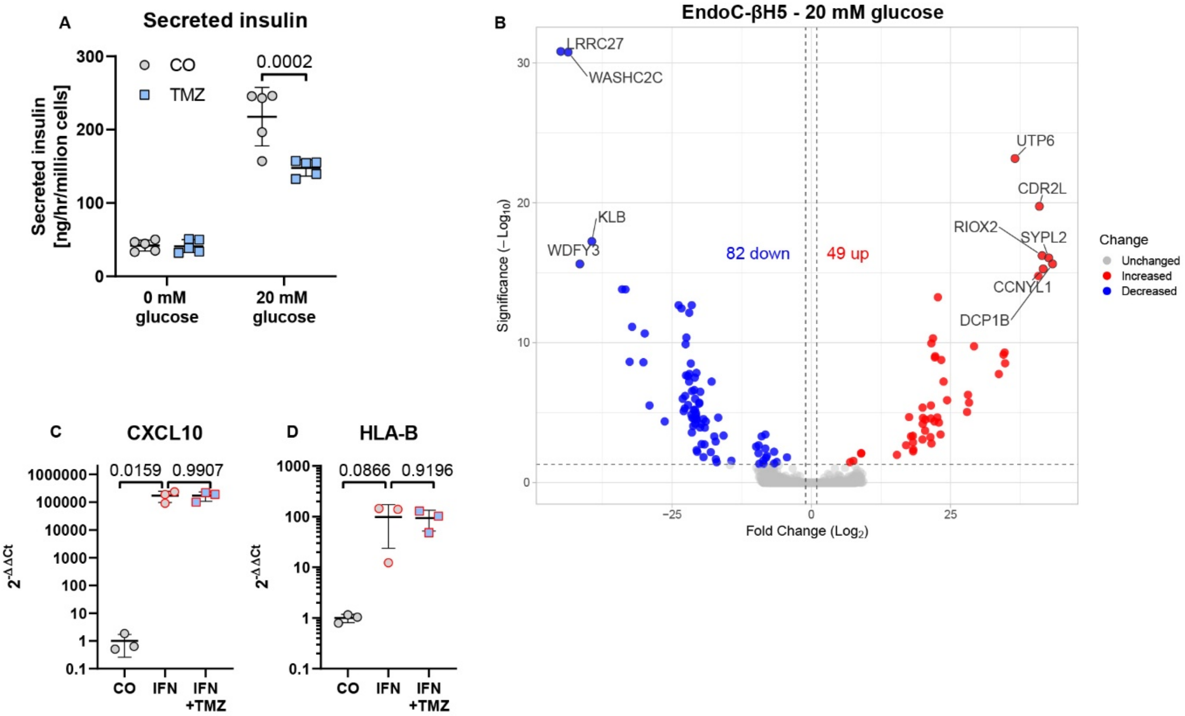

Figure S3. RNAseq of skeletal muscle cells and EndoC-βH5 cells.

(A) Glucose-stimulated insulin secretion (GSIS) in EndoC-βH5 cells after 40-minutes stimulation with 20 mM glucose and TMZ.
(B) Volcano plot of RNA-seq data from EndoC-βH5 cells treated with 20 mM glucose and TMZ for 40 minutes.
(C-D) EndoC-βH5 cells were treated with 20 ng/mL IFN-γ or IFN-γ and TMZ for 48 hours. Expression of *CXCL10* (C) and *HLA-B* (D) mRNA was quantified by qPCR.

(A) n= 5. 2way ANOVA with Sidak's multiple comparisons test was used for statistical analysis.
(B) n = 3.
(C-D) n= 3. Unpaired t-test was used for statistical analysis.

CO: Control; IFN: Interferon gamma; TMZ: Trimetazidine.

Figure S4

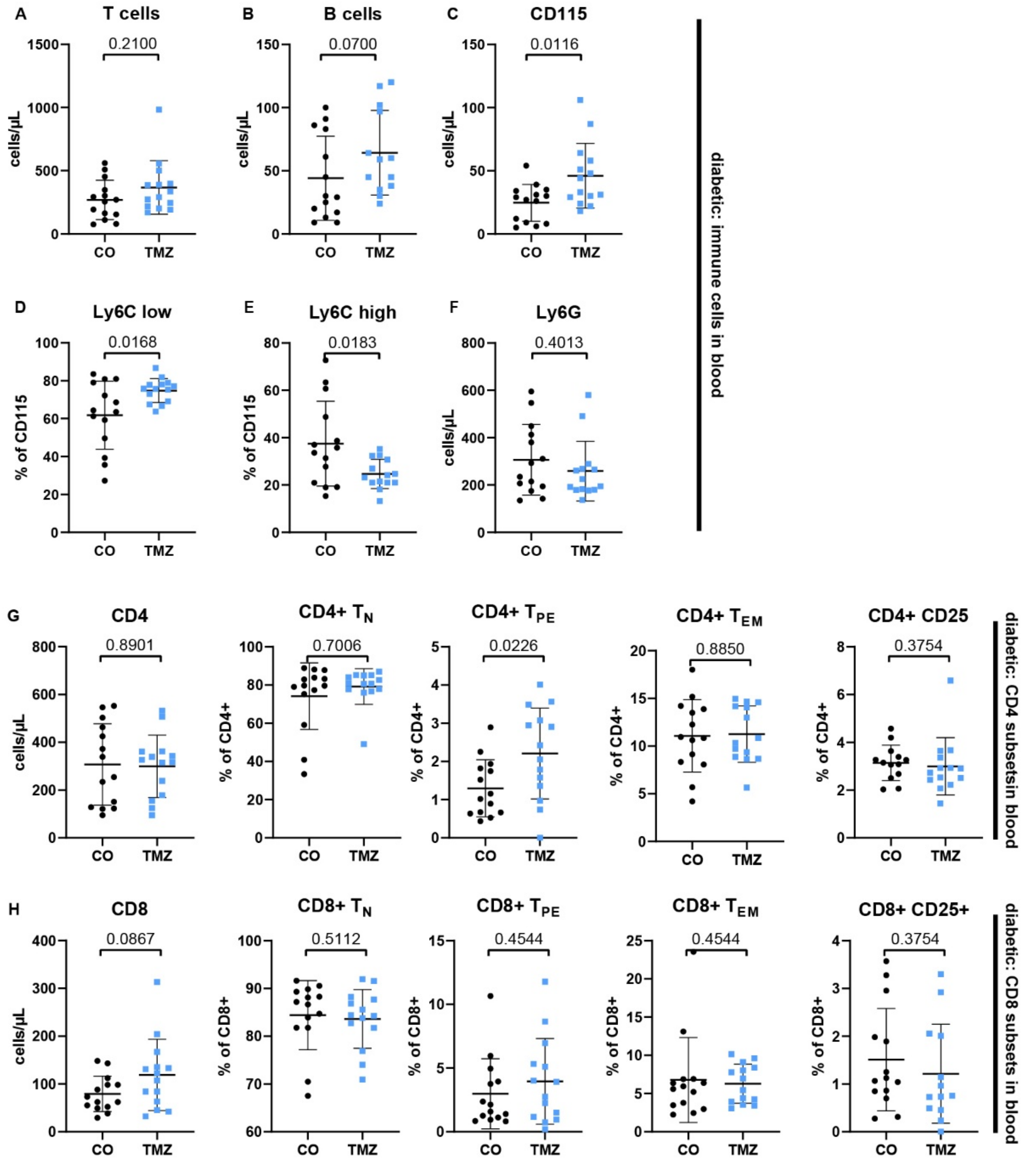

Figure S4. Flow cytometric analysis of circulating immune cell populations in NOD mice treated with Trimetazidine (TMZ)

Circulating immune cells in diabetic mice:
(A) $CD3^+$ T cells, (B) $B220^+$ B cells, (C) $CD115^+$ monocytes, (D) $Ly6C^{low}$ monocytes, (E) $Ly6C^{high}$ monocytes, (F) $Ly6G^+$ neutrophils.
(G) $CD4^+$ T cells and their subsets: T_N, T_{PE}, T_{EM}, and $CD25^+$ cells,
(H) $CD8^+$ T cells and their subsets: T_N, T_{PE}, T_{EM}, and $CD25^+$ cells.

n= 13-14. Unpaired t-test or Mann-Whitney test were used for statistical analysis.
CO: Control; TMZ: Trimetazidine; T_N: naïve T cells; T_{PE}: pre-effector T cells; T_{EM}: effector memory T cells

Figure S5

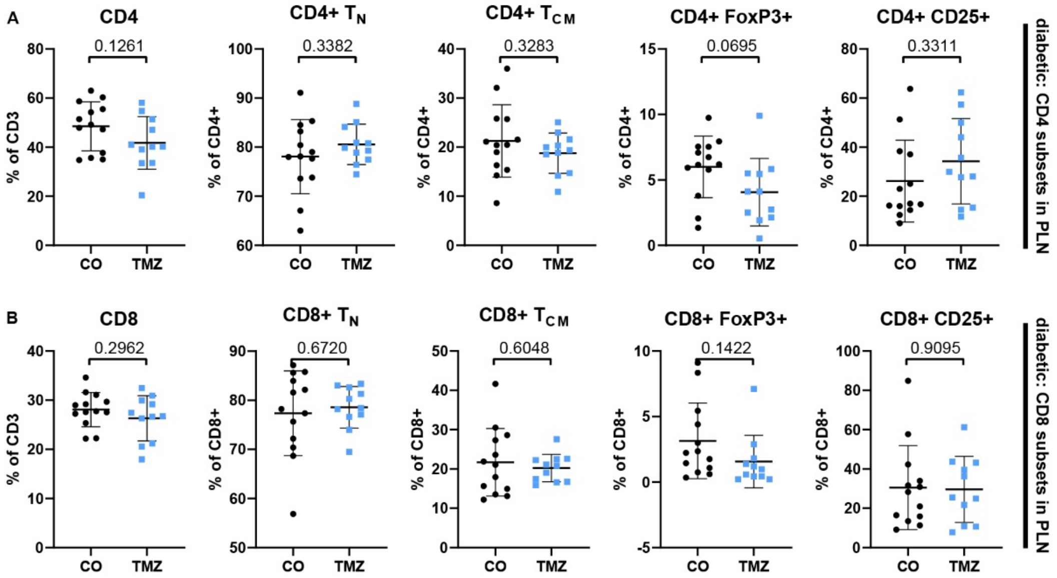

Figure S5. Flow cytometric analysis of lymphoid immune cell populations in NOD mice treated with Trimetazidine (TMZ)

Pancreatic lymph node T cells in diabetic mice:
(A) $CD4^+$ T cells and their subsets: T_N, T_{CM}, $FoxP3^+$ regulatory T cells, and $CD25^+$ T cell,
(B) $CD8^+$ T cells and their subsets: T_N, T_{CM}, $FoxP3^+$ regulatory T cells, and $CD25^+$ T cell.

n= 13-14.
Unpaired t-test or Mann-Whitney test were used for statistical analysis.
CO: Control; TMZ: Trimetazidine; T_N: naïve T cells; T_{CM}: central memory T cells

Table S1

20 mM Glucose: Trimetazidine vs Control	NES	FDR q-val
GOBP_SENSORY_PERCEPTION_OF_SMELL	1.5961068	0.16712083
REACTOME_SCAVENGING_OF_HEME_FROM_PLASMA	1.5810477	0.17482255

Table S1. Gene set enrichment analysis (GSEA) of glucose-stimulated EndoC-βH5 cells treated with Trimetazidine (TMZ).

GSEA was performed on RNA-seq data from EndoC-βH5 cells treated with 20 mM glucose and either vehicle or TMZ for 40 minutes. Shown are gene sets with normalized enrichment scores (NES) and corresponding false discovery rate (FDR q-values). Only pathways with FDR < 0.25 are considered significant.

Figure S6

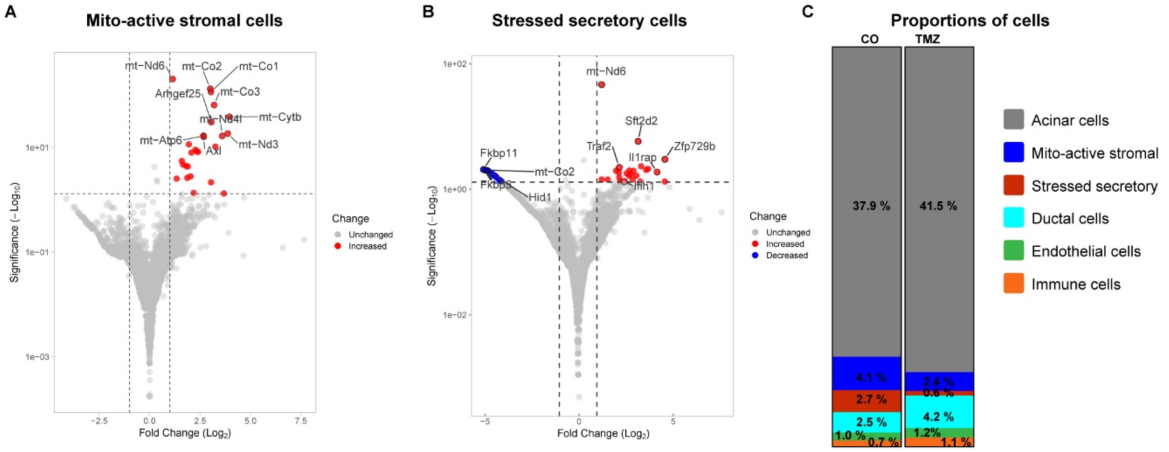

Figure S6. UMAP-based clustering and differential expression of mitochondrially active stromal and stressed secretory cells.
(A) Uniform Manifold Approximation and Projection (UMAP) of scRNA-seq data showing all pancreatic cells across control and TMZ-treated conditions. Six major cell clusters are annotated: acinar cells, mitochondrially active stromal cells, stressed secretory cells, ductal cells, endothelial cells, and immune cells. Representative defining genes are labeled adjacent to each cluster using corresponding color codes.
(A) Volcano plot of differential gene expression between mitochondrially active stromal cells and all other cell types.
(B) Volcano plot of differential gene expression between stressed secretory cells and all other cell types.
(C) Proportions of cellular clusters, identified with single cell RNA sequencing of pancreatic tissue from mice with a blood glucose >150 mg/dL and TMZ or control treatment for 1 week.

CO: Control; TMZ: Trimetazidine.

Figure S7

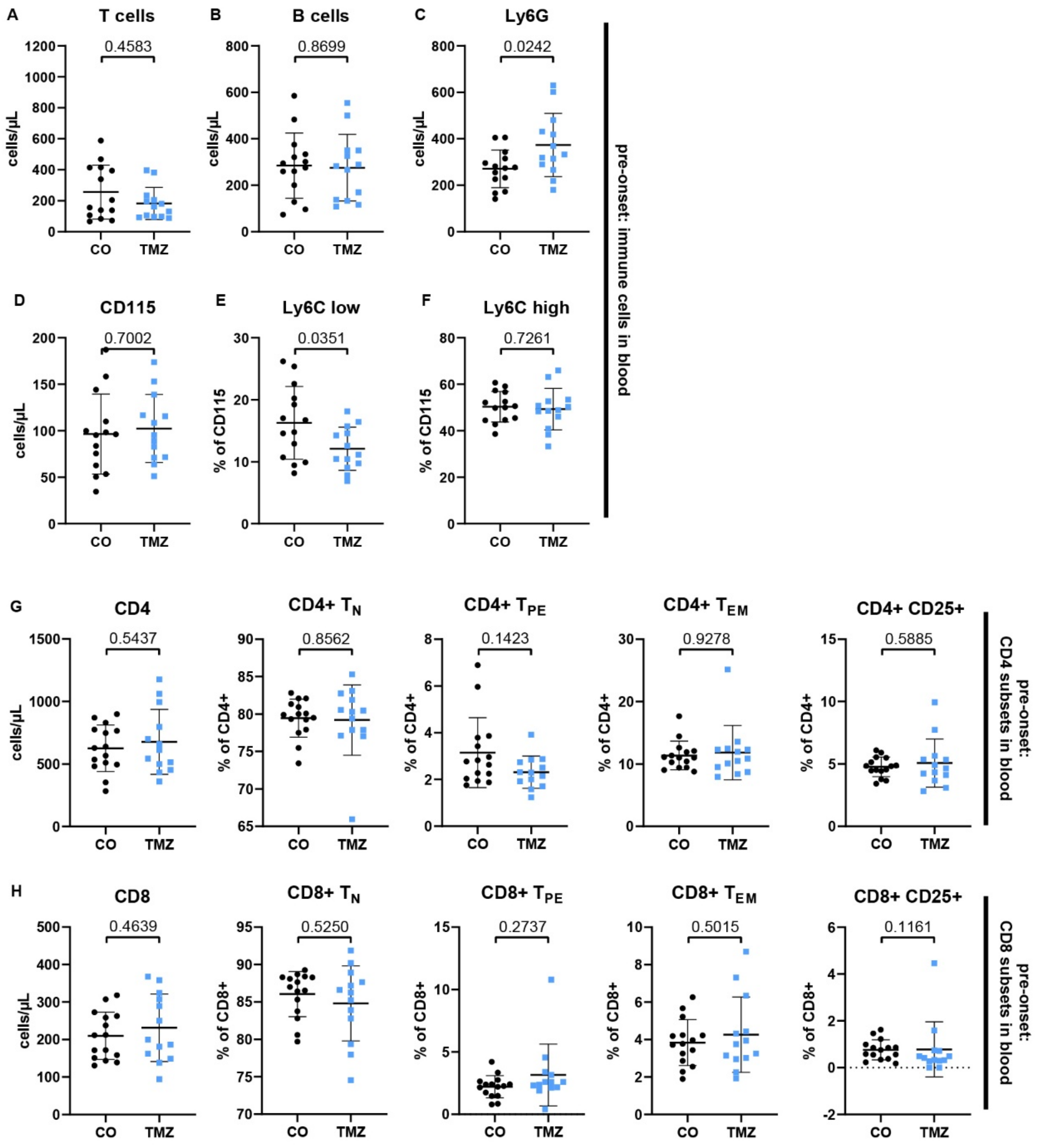

Figure S7. Peripheral immune profiling in mice prior to diabetes onset following prophylactic Trimetazidine treatment

Flow cytometric analysis of circulating immune cells in mice prior to diabetes onset (15 weeks of age) after prophylactic TMZ treatment:

(A) CD3$^+$ T cells, (B) B220$^+$ B cells, (C) Ly6G$^+$ neutrophils (D) CD115$^+$ monocytes, (E) Percentage of Ly6Cl^{ow} CD115$^+$ monocytes, (F) Percentage of Ly6C^{high} CD115$^+$ monocytes,

(G) CD4$^+$ T cells and their subsets: T_N, T_{PE}, T_{EM}, and CD25$^+$ cells,

(H) CD8$^+$ T cells and their subsets: T_N, T_{PE}, T_{EM}, and CD25$^+$ cells.

(A-F) n= 13-14; (G,H) n= 13-15

Unpaired t-test or Mann-Whitney test were used for statistical analysis.

CO: Control; TMZ: Trimetazidine; T_N: naïve T cells; T_{PE}: pre-effector T cells; T_{EM}: effector memory T cells

Figure S8

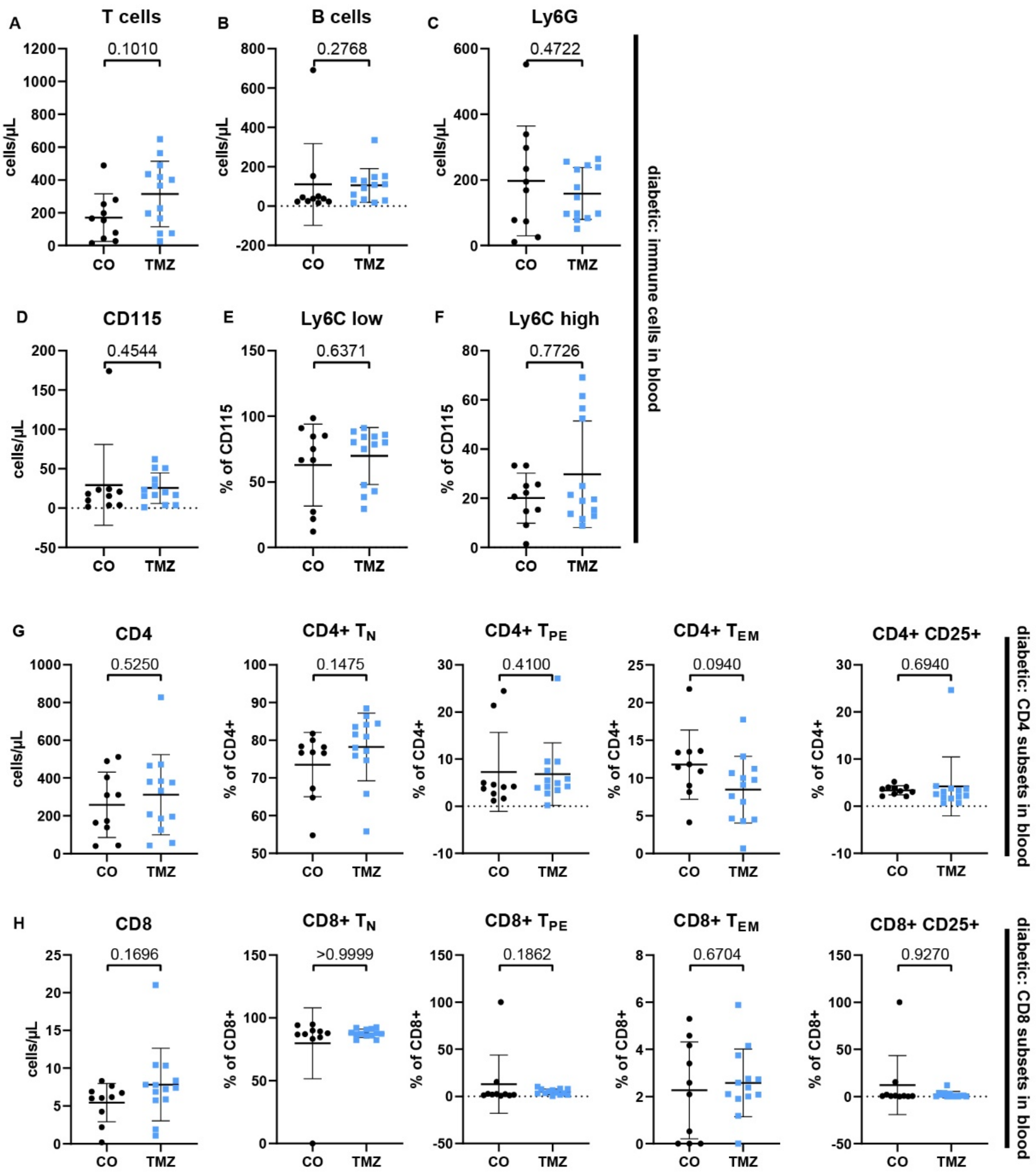

Figure S3. Peripheral immune profiling in diabetic mice following prophylactic Trimetazidine treatment

Flow cytometric analysis of circulating immune cells in diabetic mice after long-term TMZ treatment:

(A) CD3$^+$ T cells, (B) B220$^+$ B cells, (C) CD115$^+$ monocytes, (D) Percentage of Ly6Cl^{ow} CD115$^+$ monocytes, (E) Percentage of Ly6C^{high} CD115$^+$ monocytes, (F) Ly6G$^+$ neutrophils.

(G) CD4$^+$ T cells and their subsets: T_N, T_{PE}, T_{EM}, and CD25$^+$ cells.

(H) CD8$^+$ T cells and their subsets: T_N, T_{PE}, T_{EM}, and CD25$^+$ cells.

n= 10-13

Unpaired t-test or Mann-Whitney test were used for statistical analysis.

CO: Control; TMZ: Trimetazidine; T_N: naïve T cells; T_{PE}: pre-effector T cells; T_{EM}: effector memory T cells

Figure S9

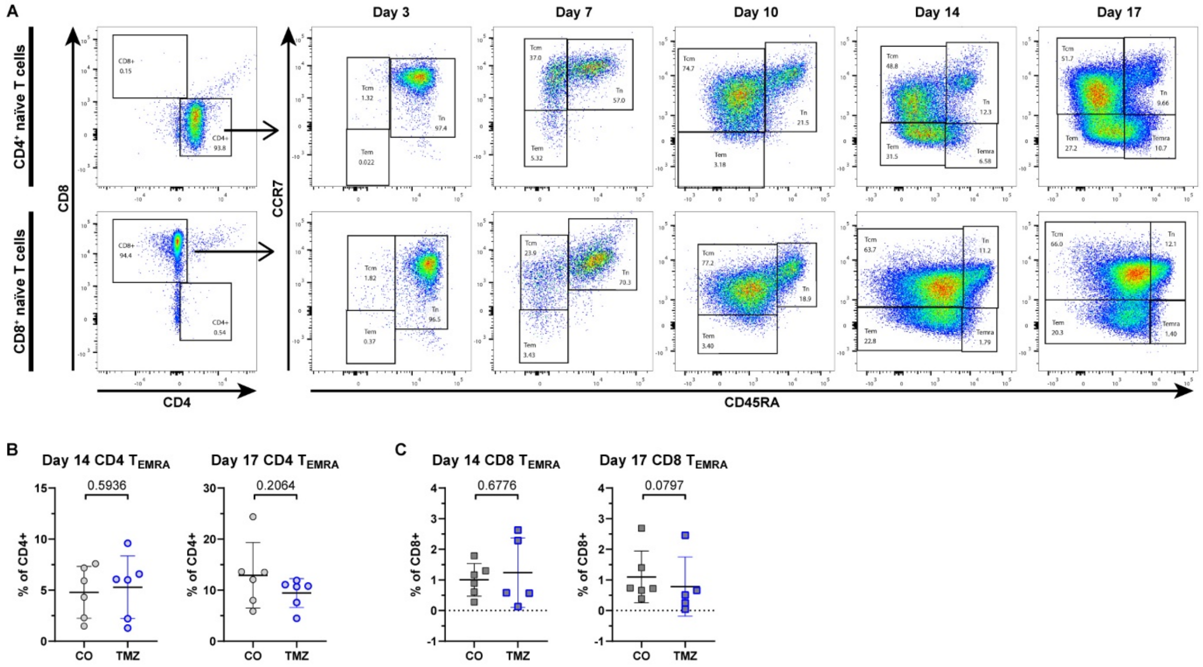

Figure S9. Gating strategy and CD4$^+$/CD8$^+$ TEMRA differentiation profiles during in vitro TMZ treatment.

(A) Representative gating strategy for identifying CD4$^+$ (top row) and CD8$^+$ (bottom row) T cell subsets based on CCR7 and CD45RA expression. Shown are days 3, 7, 10, 14, and 17. Naïve (T_N; CCR7$^+$CD45RA$^+$), central memory (T_{CM}; CCR7$^+$CD45RA$^-$), effector memory (T_{EM}; CCR7$^-$CD45RA $^-$), and Terminally differentiated effector memory T cells re-expressing CD45RA (T_{EMRA}; CCR7$^-$CD45RA$^+$) populations were quantified.

(B) Representative dot plots showing T_{EMRA} subset frequencies in CD4$^+$ T cells on days 14 and 17.

(C) Representative dot plots showing T_{EMRA} subset frequencies in CD8$^+$ T cells on days 14 and 17.

n = 5-6 human donors with or without TMZ treatment. Paired t-test was used for statistical analysis. CO: Control; TMZ: Trimetazidine; T_N: naïve T cells; T_{CM}: central memory T cells; T_{EM}: effector memory T cells; T_{EMRA}: terminally differentiated effector memory T cells re-expressing CD45RA

Figure S10

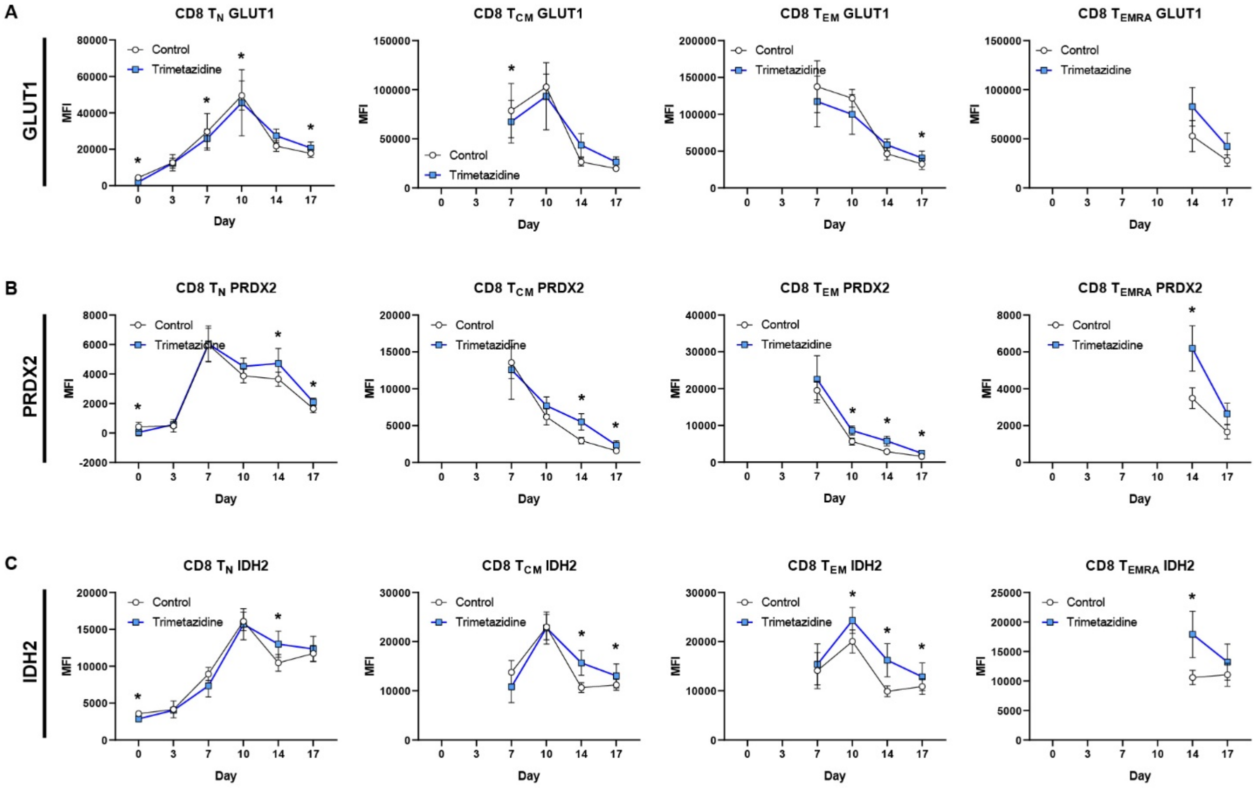

Figure S10. Complementary metabolic adaptations in CD8⁺ T cells after Trimetazidine treatment

Naïve human CD8⁺ T cells were cultured under stimulatory conditions with or without TMZ for 17 days. Temporal expression patterns of GLUT1 (A), PRDX2 (B), and IDH2 (C) across CD8⁺ T cell subsets from day 0 to 17.

n = 5-6 human donors with or without TMZ treatment. Paired t-test was used for statistical analysis. CO: Control; TMZ: Trimetazidine; T_N: naïve T cells; T_{CM}: central memory T cells; T_{EM}: effector memory T cells; T_{EMRA}: terminally differentiated effector memory T cells re-expressing CD45RA

Figure S11

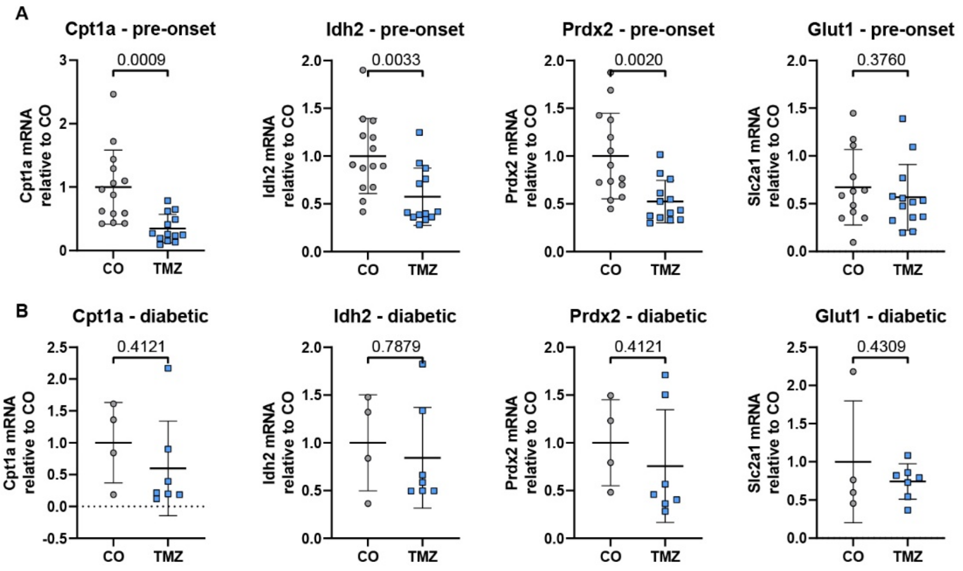

Figure S11. Pancreatic mRNA expression of Cpt1a, Idh2, Prdx2, and Slc2a1

(A) Pancreatic mRNA expression of *Cpt1a, Idh2, Prdx2,* and *Slc2a1* (*Glut1*) in pre-onset NOD mice after 10 weeks of prophylactic TMZ treatment, quantified by qPCR. n = 13-14

(B) Pancreatic mRNA expression of *Cpt1a, Idh2, Prdx2,* and*, Slc2a1* (*Glut1*) in diabetic NOD mice after 10 weeks of prophylactic TMZ treatment, quantified by qPCR.

n = 4-7 mice. Unpaired t-test or Mann-Whitney test were used for statistical analysis.

CO: Control; TMZ: Trimetazidine

Figure S12

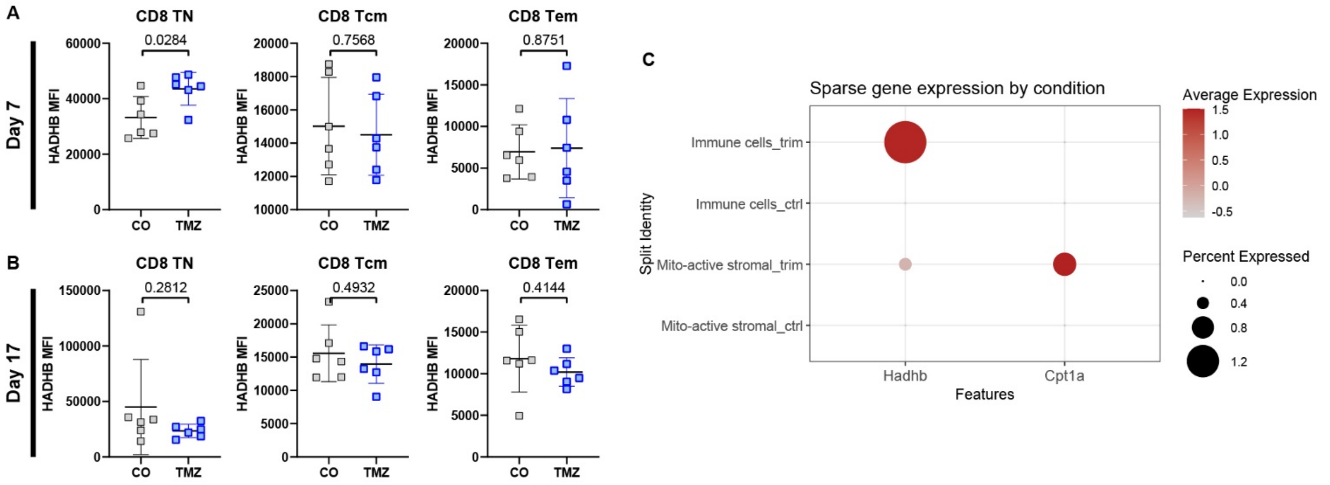

Figure S12. Fatty acid oxidation adaptations after Trimetazidine treatment

(A-B) Naïve human CD8^{+} T cells were cultured under stimulatory conditions with or without TMZ for 17 days. Temporal expression patterns of HADHB across CD8^{+} T cell subsets at (A) day 7 and (B) day 17.

(C) scRNA-seq data from pre-diabetic mice: Gene expression of Hadhb and Cpt1a 7 in mitochondrially active stromal cells and immune cells.

n = 6 human donors with or without TMZ treatment. Paired t-test was used for statistical analysis. CO: Control; TMZ: Trimetazidine; HADHB: 3-ketacyl-CoA thiolase; T_N: naïve T cells; T_{CM}: central memory T cells; T_{EM}: effector memory T cells

Figure S13

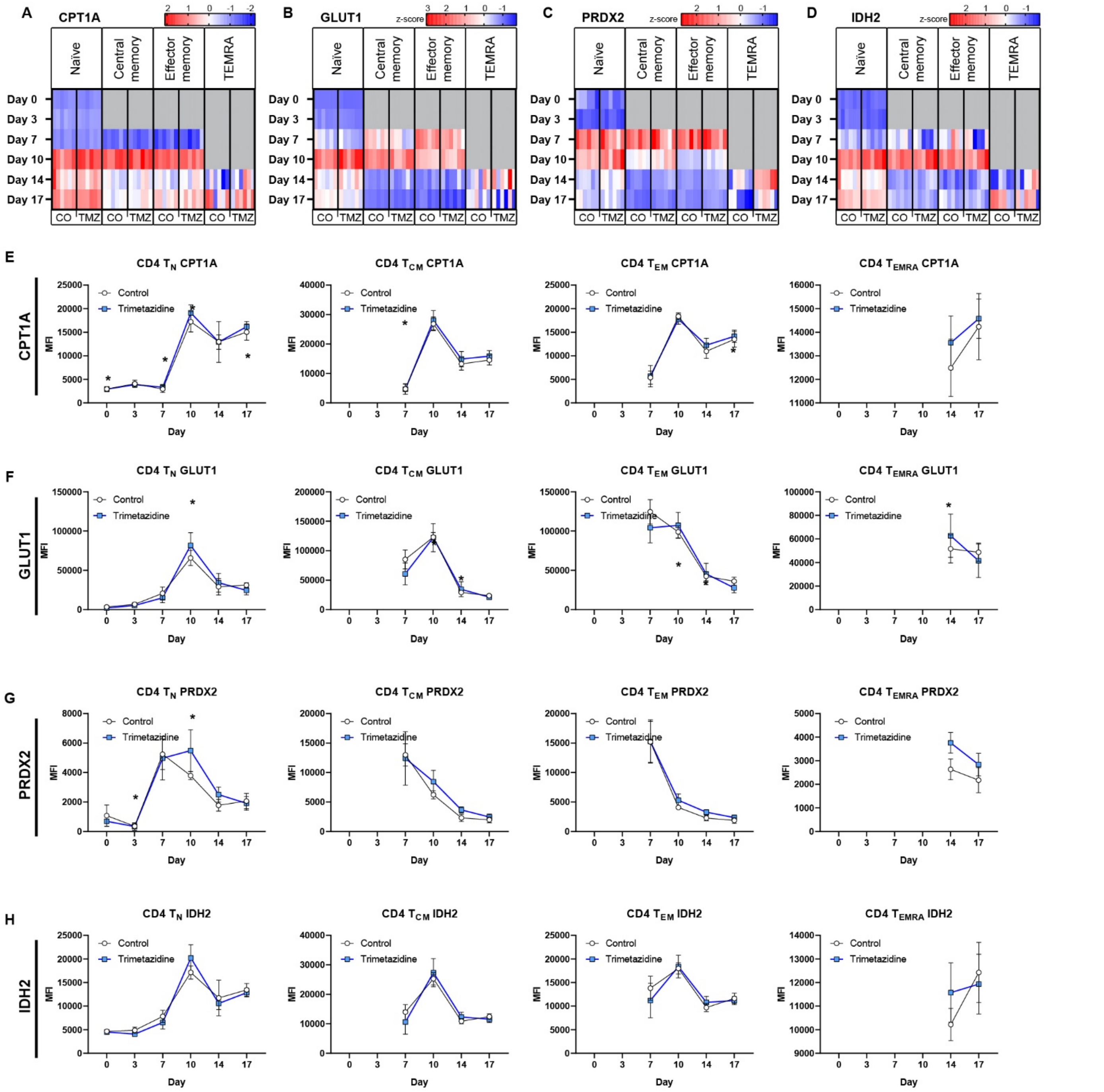

Figure S13. Complementary metabolic adaptations in CD4$^+$ T cells after Trimetazidine treatment

Naïve human CD4$^+$ T cells were cultured under stimulatory conditions with or without TMZ for 17 days. (A-D) Heatmaps of z-scored expression levels for CPT1A (A), GLUT1 (B), PRDX2 (C), and IDH2 (D) in CD4$^+$ TN, TCM, TEM, and TEMRA subsets across all time points.

(E-H) Time-course quantification of CPT1A (E), GLUT1 (F), PRDX2 (G), and IDH2 (H) expression in CD4$^+$ TN, TCM, TEM, and TEMRA subsets.

n = 5-6 human donors with or without TMZ treatment. Paired t-test was used for statistical analysis. CO: Control; TMZ: Trimetazidine; T_N: naïve T cells; T_{CM}: central memory T cells; T_{EM}: effector memory T cells; T_{EMRA}: terminally differentiated effector memory T cells re-expressing CD45RA
